# Inborn errors of metabolism in neonates and pediatrics on varying dialysis modalities: a systematic review and meta-analysis

**DOI:** 10.1007/s00467-024-06547-7

**Published:** 2024-11-11

**Authors:** Manan Raina, Kush Doshi, Archana Myneni, Abhishek Tibrewal, Matthew Gillen, Jieji Hu, Timothy E. Bunchman

**Affiliations:** 1 Hawken High School, Cleveland, OH USA; 2 Akron Nephrology Associates/Cleveland Clinic Akron General Medical Center, Akron, OH USA; 3 Division of Neonatal-Perinatal Medicine, Department of Pediatrics, Emory University School of Medicine, Atlanta, GA USA; 4 College of Medicine, Northeast Ohio Medical University, Rootstown, OH USA; 5 Department of Pediatric Nephrology & Transplantation, Children’s Hospital of Richmond at the Virginia Commonwealth University, 1000 E Broad St, PO Box 980498, Richmond, VA 23298 USA

**Keywords:** IEM, Neonates, Pediatrics, CKRT

## Abstract

**Background:**

Some inborn errors of metabolism (IEMs) resulting in aberrations to blood leucine and ammonia levels are commonly treated with kidney replacement therapy (KRT). Children with IEMs require prompt treatment, as delayed treatment results in increased neurological and developmental morbidity.

**Objectives:**

Our systematic review in neonates and pediatrics evaluates survival rates and reductions in ammonia and leucine levels across different KRT modalities (continuous KRT (CKRT), hemodialysis (HD), peritoneal dialysis (PD)).

**Data sources:**

A literature search was conducted through PubMed, Web of Science, and Embase databases for articles including survival rate and toxic metabolite clearance data in pediatric patients with IEM undergoing KRT.

**Study eligibility criteria:**

Cross-sectional, prospective, and retrospective studies with survival rates reported in patients with IEM with an intervention of CKRT, PD, or HD were included. Studies with patients receiving unclear or multiple KRT modalities were excluded.

**Study appraisal and synthesis methods:**

Analysis variables included efficacy outcomes [% reduction in ammonia (RIA) from pre- to post-dialysis and time to 50% RIA] and mortality. The Newcastle Ottawa Risk of Bias quality assessment was used to assess bias. All statistical analyses were performed with MedCalc Statistical Software version 19.2.6.

**Results:**

A total of 37 studies (*n* = 642) were included. The pooled proportion (95% CI) of mortality on CKRT was 24.84% (20.93–29.08), PD was 34.42% (26.24–43.33), and HD 34.14% (24.19–45.23). A lower trend of pooled (95% CI) time to 50% RIA was observed with CKRT [6.5 (5.1–7.8)] vs. PD [14.4 (13.3–15.5)]. A higher mortality was observed with greater plasma ammonia level before CKRT (31.94% for ≥ 1000 µmol/L vs. 15.04% for < 1000 µmol/L).

**Conclusions and implications of key findings:**

Despite the limitations in sample size, trends emerged suggesting that CKRT may be associated with lower mortality rates compared to HD or PD, with potential benefits including prevention of rebound hyperammonemia and improved hemodynamic control. While HD showed a trend towards faster achievement of 50% RIA, all modalities demonstrated comparable efficacy in reducing ammonia and leucine levels.

**Prospero registration:**

CRD42023418842.

**Graphical abstract:**

**Figure:**
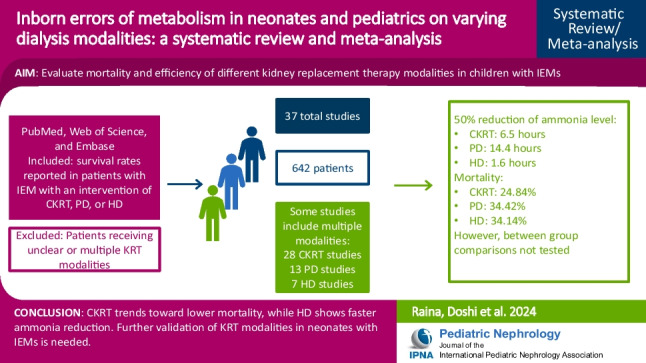
A higher resolution version of the Graphical abstract is available as Supplementary information

**Supplementary Information:**

The online version contains supplementary material available at 10.1007/s00467-024-06547-7.

## Introduction

Inborn errors of metabolism (IEMs) are a heterogeneous group of disorders due to single gene defects affecting enzymes that facilitate the conversion of substrates into metabolites. These disorders involve aberrations in the metabolism of macromolecules (i.e., carbohydrates, fats, or proteins). Complications of IEM arise due to the accumulation of toxic substances without adequate removal or excretion, including ammonia, leucine, and other related metabolites [1]. IEMs that cause hyperammonemia include urea cycle disorders (UCD), organic acidemias (OA), fatty acid oxidation disorders (FAOD), and amino acidopathies [2]. Maple syrup urine disease (MSUD) is a type of OA that presents in four distinct types: classical, intermediate, intermittent, and thiamine-responsive [3].

Ammonia is produced by catabolism of amino acids, deamination of adenosine monophosphate, or via the glutamine–glutamate cycles in the liver, kidney, pancreas, and brain. Hyperammonemia is the toxic accumulation of ammonia, which leads to a constellation of symptoms, including poor feeding, lethargy, apnea, seizures, encephalopathy, edema, coma, and death [4]. For children with MSUD, increased concentrations of branched-chain amino acids and branched-chain α-ketoacids contribute to neurotoxicity [3]. Diminished or absent activity of the branched-chain α-ketoacid dehydrogenase complex predominantly affects the central nervous system, leading to cerebral edema, coma, and neurological impairments [3]. Suspicion for an IEM is based upon detecting abnormal metabolite levels, which can often require 24–72 h for a biochemical diagnosis, further delaying treatment. Persistently elevated toxic metabolite levels are especially concerning as the duration of untreated IEMs is shown to correlate with worsening neurological outcomes and developmental sequelae [5, 6]. The onset of symptoms for IEMs is variable, as complete loss of enzyme activity leads to severe presentation in the neonatal period. In contrast, partial dysfunction leads to definitive clinical presentation in infancy and childhood. Therefore, treatment of IEMs is a medical emergency, especially in neonates and children, thus necessitating empiric treatment.

Management of an IEM depends on its underlying etiology. If toxic metabolite concentrations are refractory to diet and pharmacological therapies, kidney replacement therapy (KRT), such as hemodialysis (HD), peritoneal dialysis (PD), or continuous kidney replacement therapy (CKRT), should be started immediately [2]. Initial studies by Donn et al. compared exchange transfusion, PD, and HD, and concluded that HD is the superior modality for treating hyperammonemia secondary to a UCD [7]. Intermittent hemodialysis (IHD) may provide the highest metabolite extraction due to the relationship between solute clearance, dialysate flow rate, blood flow rate, and the surface area of the dialytic membrane, but its feasibility can be limited by technical and hemodynamic complications [8]. CKRT may be preferable for continuous, well-tolerated ammonia clearance as first-line therapies in young infants [9]. PD is slower at removing metabolite levels but should be considered if HD and CKRT are not feasible. A thorough and comparative analysis regarding efficacy and mortality rates of differing KRT as an option for acute extracorporeal detoxification in the pediatric population is needed. Our review aims to comprehensively analyze the efficacy and mortality rates of varying KRT modalities utilized in neonatal and pediatric patients with IEM.

## Methods

### Literature search

Two independent reviewers searched PubMed, Web of Science, and Embase. Search Strategies were split into three searches (one for each mode of dialysis) to account for more relevant results. Primary Search Queries for the first search included the terms “Inborn errors of Metabolism, IEM, hyperammonemia, urea cycle disorder, pediatrics neonates, CKRT, continuous renal replacement therapy.” For the peritoneal dialysis search, identical queries were used except for substituting the “CKRT” for “peritoneal dialysis OR PD”. Likewise, “Hemodialysis OR HD” was utilized for the third search. The search was restricted to pediatric/neonate age groups and the English language. There were no limitations on publication year and published studies ranged from 1999–2024. Selected articles were exported to a citation managing software, “Rayyan”. Two independent reviewers, MR and KD, reviewed all results based on predefined inclusion and exclusion criteria. Studies that met the inclusion criteria were retrieved for full-text review. The quality of the included studies was assessed using the Newcastle–Ottawa Risk of Bias quality assessment. Any disagreements between the two reviewers during the screening process were resolved through discussion. In cases where a consensus could not be reached, a third reviewer, TB, resolved any disagreements regarding data extraction. A detailed search strategy is outlined in Supplemental Table S1.

### Selection criteria

Studies selected for inclusion were those with survival rates reported in patients with IEM with an intervention of CKRT, PD, or HD. Studies were not restricted by geographic location. This study did not include articles not in English, systematic reviews, or self-reported outcomes. Cross-sectional, prospective, and retrospective studies were included in the review to assess the variable difference in ammonia levels in patients with IEM before and after KRT, and their respective survival rates. Patients receiving unclear or multiple KRT modalities were excluded. The complete inclusion and exclusion criteria are outlined in Supplemental Table S2.

### Data extraction

Standardized data collection forms comprised the first author, title, year of publication, sample size, age at admission or KRT initiation, geography, and study design. Patient-specific characteristics included IEM etiology, dialysis mode, indications for KRT, ammonia levels, leucine levels, KRT prescriptions, and survival outcomes. Studies can be found in Supplemental Table S3 [10–46].

### Statistical analysis

The analysis included a decrease in ammonia/leucine level pre- and post- dialysis, time to 50% reduction in ammonia level, and mortality among children with IEMs on different dialysis modalities (CKRT, PD or HD). The pooled decrease in ammonia/leucine level, time to 50% reduction in ammonia level, and mortality with 95% CIs have been reported for the different dialysis modalities. The mortality across the dialysis modalities has been compared using the pooled odds ratio (95% CI). Heterogeneity across studies was quantified using the I^2^ statistic, and the I^2^ > 50% indicated significant heterogeneity. To determine the source of heterogeneity, sensitivity analyses were performed based on these parameters (sample size, year of the study, neonate vs. non-neonate). The fixed-effect analytical model was used to pool the results of studies with acceptable or no heterogeneity, and the random-effect model was used for studies with significant heterogeneity. A forest plot was used to visualize the mortality outcome in each study and the combined estimated outcome with their 95% CI. Publication bias was assessed graphically using funnel plots and Egger’s test. A *p*-value ≤ 0.05 was considered for statistical significance. All statistical analyses were performed with MedCalc Statistical Software version 19.2.6 (MedCalc Software bv Ostend, Belgium; https://www.medcalc.org; 2020).

## Results

### Included studies

A total of 37 studies (only CKRT (18 studies), only PD (5 studies), only HD (4 studies), both CKRT and PD (7 studies), both CKRT and HD (2 studies), both PD and HD (0 study) and all three modalities (1 study)) were included. Of these 37 studies, 24 were conducted in neonates and 13 in non-neonates. The total sample size of all the included studies was 642, ranging from 2 to 145 across the studies. The mean/median age at admission ranged from 3–12 days across 12 studies in neonates and from 2.5 to 7.5 years across six studies in non-neonates. The mean/median age at dialysis ranged from 3–15 days across 11 studies in neonates and from 2 months to 6.5 years across five studies in non-neonates. The time to KRT initiation from symptom onset ranged from 6–26 h across three studies reporting the data. The majority of the studies were conducted in Eurasia (Turkey; *n* = 13), followed by 11 in Europe (Germany, France, Italy, UK, Slovenia), 8 in Asia (China, Korea, Japan, Taiwan, Israel), 4 in North America (US or Canada) and 1 in South America (Chile). 18 studies included mention of using metabolic scavengers and/or nutritional supplementation to treat patients prior to initiation of KRT, while pre-dialysis treatments were not mentioned in the remainder of the studies.

### Ammonia and leucine clearance in CKRT studies

Among studies with CKRT, the number of included studies was 28, with a total of 441 CKRT patients with a sample size ranging from 2 to 137 patients across the studies. The mean/median duration of CKRT ranged from 16–120 h across the 14 studies reporting the data. In the 31 studies that included CKRT, the blood flow ranged from 5–75 mL/min, while the dialysate flow ranged from 2,000–12,900 mL/1.73 m^2^/hour. The mean/median plasma ammonia before CKRT ranged from 391–2,132 µmol/L, while after CKRT it ranged from 29–228 µmol/L across the six studies reporting the data. The pooled mean (95% CI) plasma ammonia level before CKRT was 1,052 (737–1,367) µmol/L [I^2^ (95% CI): 96.39% (94.19–97.76), *p* < 0.0001, 6 studies, *n* = 101] and at the end of CKRT was 84.7 (49.5–119.9) µmol/L [I^2^: 94.44% (89.8–96.96), *p* < 0.0001, 6 studies, *n* = 101], for an average ammonia reduction of 91.9% (95% CI: 88.60–95.29, *p* < 0.0001). The pooled (95% CI) time to 50% reduction of ammonia level among those with CKRT was 6.5 (5.1–7.8) hours [I^2^: 85.4% (73.2–92.1), *p* < 0.0001, 8 studies, *n* = 87]. The pooled mean (95% CI) plasma leucine level before CKRT was 1,696.3 (1,389.7–2,002.9) µmol/L [I^2^ (95% CI): 0.00% (0.00–0.00), *p* = 0.4011, two studies, *n* = 22] and at the end of CKRT was 311.7 (109.7–513.6) µmol/L [I^2^: 97.76% (95.76–98.82), *p* < 0.0001, 3 studies, *n* = 33]. The Egger’s test (*p* > 0.05) for all the above-pooled analyses except for pre-treatment leucine level indicated the absence of publication bias.

### Ammonia and leucine clearance in PD studies

Among studies with PD, the number of included studies was 13, with a total of 123 PD patients with a sample size ranging from 1 to 29 patients across the studies. The mean/median duration of PD ranged from 60–110 h across the four studies reporting the data. The duration required to decrease plasma ammonia < 200 μg/dL was around 36 h in 3 studies [27, 29, 30]. The pooled (95% CI) time to 50% reduction of ammonia level among those with PD was 14.4 (13.3–15.5) hours [I^2^:17.69% (0.00–61.53), *p* = 0.2949, seven studies, *n* = 91]. The mean (SD) plasma leucine concentration pre-dialysis was 3,117 (873) µmol/L in one study while the pooled mean (95% CI) plasma leucine concentration post-dialysis was 803.0 (712.7 – 893.4) µmol/L [I^2^ (95% CI): 0.00% (0.00–0.00), *p* = 0.3835, two studies, *n* = 14] [22].

### Ammonia and leucine clearance in HD studies

Among studies with HD, the number of included studies was 7, with a total of 78 HD patients with a sample size ranging from 2 to 29 patients across the studies. The data on mean duration of dialysis, change in ammonia/leucine level, or time to median decline was reported in only 2 studies, limiting the pooled analysis. The mean (SD) time to 50% decline in the ammonia level was 1.6 (0.4) hours [26]. The mean ammonia levels decreased by 86.5% (from 955 (444) to 129 (55) μmol/L) in two consecutive HD sessions among 13 patients with primary hyperammonemia and mean leucine levels decreased by 92.1% (from 2281 (631) to 179 (91) μmol/L) among seven subjects with MSUD [24]. The serum ammonia levels were observed to decrease to < 300 μmol/L within a median of 4.5 (IQR 2.9–7.0) hours and normalized within 7.3 (IQR 3.6–13.5) hours [23].

### Mortality

**Fig. 1:**
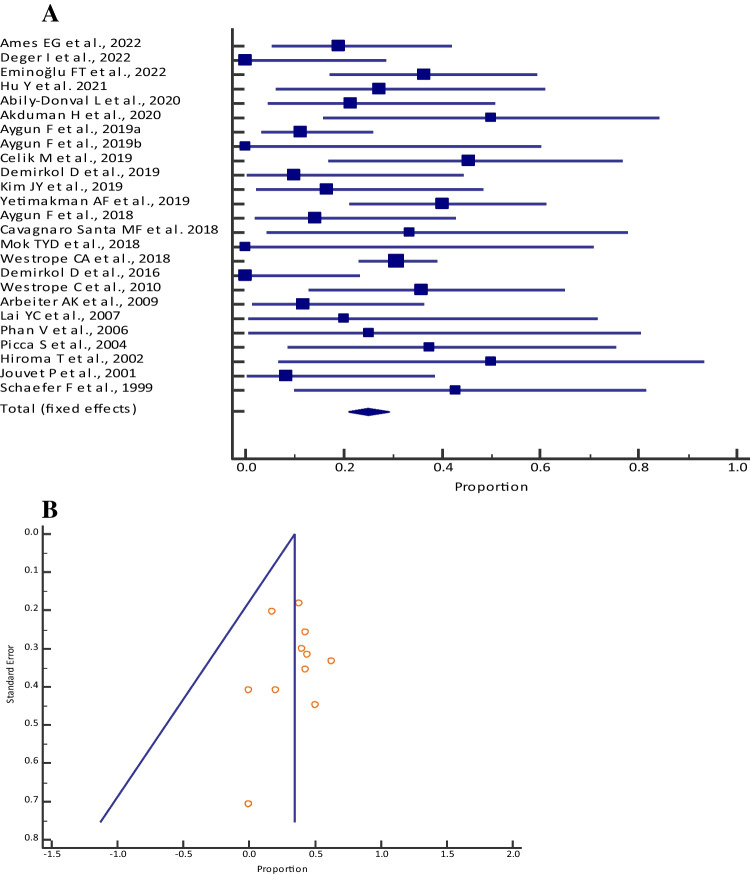
**A** Forest plot of the meta-analysis of mortality among neonates and pediatrics with inborn errors of metabolism who received continuous kidney replacement therapy across different studies. The lower diamond in the graph represents the pooled estimate. **B** Funnel plot for showing publication bias

**Fig. 2:**
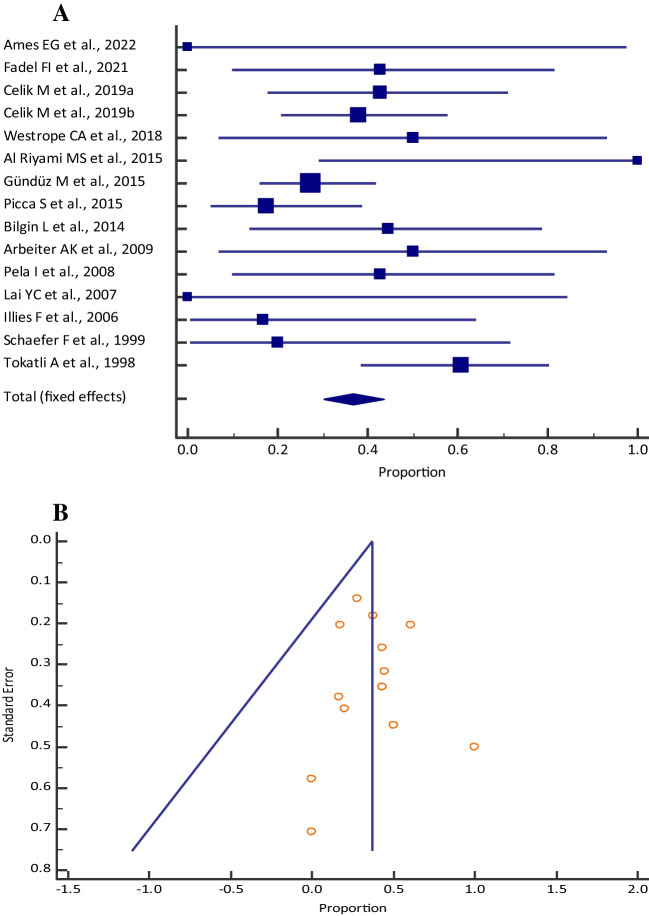
**A** Forest plot of the meta-analysis of mortality among neonates and pediatrics with inborn errors of metabolism who received peritoneal dialysis across different studies. The lower diamond in the graph represents the pooled estimate. **B** Funnel plot for showing publication bias

**Fig. 3:**
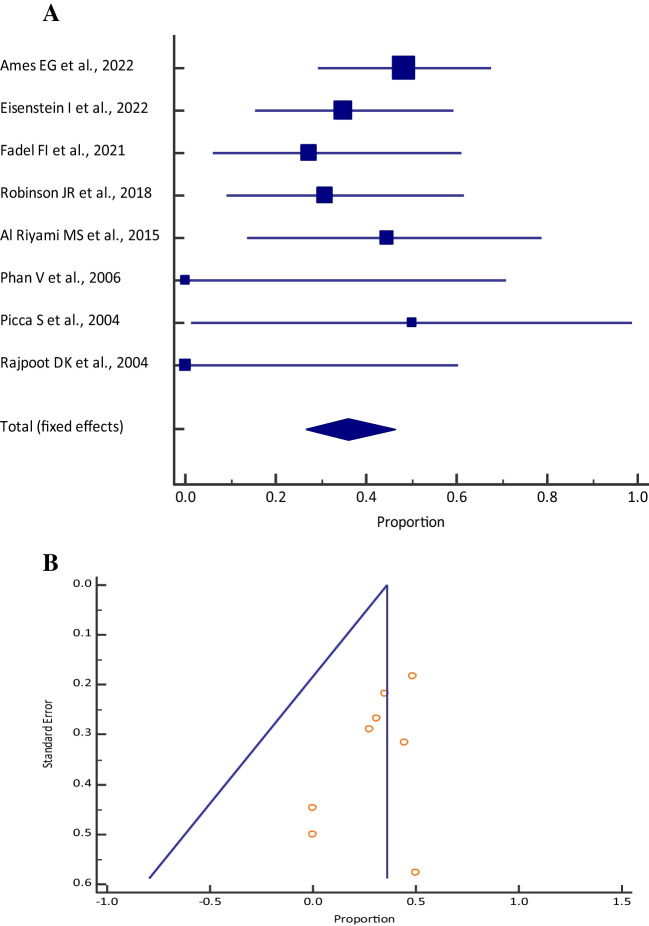
**A** Forest plot of the meta-analysis of mortality among neonates and pediatrics with inborn errors of metabolism who received hemodialysis across different studies. The lower diamond in the graph represents the pooled estimate. **B** Funnel plot for showing publication bias

The pooled proportion (95% CI) of mortality among those who received CKRT was 24.84% (20.93–29.08) [I^2^: 46.50% (14.52–66.51); *p* = 0.0061; 25 studies, fixed-effects method; *n* = 430] (Table 1, Fig. 1a), who received PD was 34.42% (26.24–43.33) [I^2^: 16.88% (0.00–56.37); *p* = 0.2784; 12 studies, fixed-effects method; *n* = 116] (Table 2, Fig. 2a) and who received HD was 34.14% (24.19–45.23) [I^2^: 34.10% (0.00–72.11); *p* = 0.1678; 7 studies, fixed-effects method; *n* = 78] (Table 3, Fig. 3a). Visual inspection of the funnel plot and Egger’s test (*p* = 0.4408, Fig. 1b; *p* = 0.7327, Fig. 2b; *p* = 0.0808, Fig. 3b) showed a symmetrical distribution, which indicated the absence of publication bias for all the different dialysis modalities.

**Table 1:** Meta-analysis of mortality among neonates with inborn errors of metabolism who received continuous kidney replacement therapy across different studies

Study	Mortality [*n*]	Sample size [*N*]	Proportion (95% CI)	Fixed weight (%)
Ames et al. 2022 [33]	4	21	19.05 (5.45–41.91)	4.84
Deger et al. 2022 [32]	0	11	0.00 (0.00–28.49)	2.64
Eminoğlu et al. 2022 [10]	8	22	36.36 (17.19–59.34)	5.05
Hu et al. 2021 [42]	3	11	27.27 (6.02–60.97)	2.64
Abily-Donval et al. 2020 [12]	3	14	21.43 (4.66–50.80)	3.30
Akduman et al. 2020 [11]	4	8	50.00 (15.70–84.30)	1.98
Aygun et al. 2019a [13]	4	36	11.11 (3.11–26.06)	8.13
Aygun et al. 2019b [35]	0	4	0.00 (0.00–60.24)	1.10
Celik et al. 2019b [27]	5	11	45.46 (16.74–76.62)	2.64
Demirkol et al. 2019 [15]	1	10	10.00 (0.25–44.50)	2.42
Kim et al. 2019 [14]	2	12	16.67 (2.09–48.41)	2.86
Yetimakman et al. 2019 [34]	10	25	40.00 (21.12–61.34)	5.71
Aygun et al. 2018 [38]	2	14	14.29 (1.78–42.81)	3.30
Cavagnaro Santa et al. 2018 [36]	2	6	33.33 (4.33–77.72)	1.54
Diane Mok et al. 2018 [16]	0	3	0.00 (0.00–70.76)	0.88
Westrope et al. 2018 [37]	42	137	30.66 (23.06–39.10)	30.33
Demirkol et al. 2016 [17]	0	14	0.00 (0.00–23.16)	3.30
Westrope et al. 2010 [18]	5	14	35.71 (12.76–64.86)	3.30
Arbeiter et al. 2010 [29]	2	17	11.77 (1.46–36.44)	3.96
Lai et al. 2007 [25]	1	5	20.00 (0.51–71.64)	1.32
Phan et al. 2006 [39]	1	4	25.00 (0.63–80.59)	1.10
Picca et al. 2001 [26]	3	8	37.50 (8.52–75.51)	1.98
Hiroma et al. 2002 [19]	2	4	50.00 (6.76–93.24)	1.10
Jouvet et al. 2001 [41]	1	12	8.33 (0.21–38.48)	2.86
Schaefer et al. 1999 [31]	3	7	42.86 (9.90–81.60)	1.76
Total (fixed effects)	108	430	24.84 (20.93–29.08)	100.00

**Table 2:** Meta-analysis of mortality among neonates with inborn errors of metabolism who received peritoneal dialysis across different studies

Study	Mortality [*n*]	Sample size [*N*]	Proportion (95% CI)	Fixed weight (%)
Ames et al. 2022 [33]	0	1	0.00 (0.00–97.50)	1.56
Deger et al. 2022 [32]	0	5	0.00 (0.00–52.18)	4.69
Celik et al. 2019a [20]	6	14	42.86 (17.66–71.14)	11.72
Celik et al. 2019b [27]	11	29	37.93 (20.68–57.74)	23.44
Westrope et al. 2018 [37]	5	8	62.50 (24.48–91.48)	7.03
Picca et al. 2015 [21]	4	23	17.39 (4.95–38.78)	18.75
Bilgin et al. 2014 [22]	4	9	44.44 (13.70–78.80)	7.81
Unal et al. 2012 [44]	4	10	40.00 (12.15–73.76)	8.59
Arbeiter et al. 2010 [29]	2	4	50.00 (6.76–93.24)	3.91
Pela et al. 2008 [30]	3	7	42.86 (9.90–81.60)	6.25
Lai et al. 2007 [25]	0	1	0.00 (0.00–97.50)	1.56
Schaefer et al. 1999 [31]	1	5	20.00 (0.51–71.64)	4.69
Total (fixed effects)	40	116	34.42 (26.24–43.33)	100

**Table 3:** Meta-analysis of mortality among neonates with inborn errors of metabolism who received hemodialysis across different studies

Study	Mortality [*n*]	Sample size [*N*]	Proportion (95% CI)	Fixed weight (%)
Ames et al. 2022 [33]	14	29	48.28 (29.44–67.47)	35.29
Eisenstein et al. 2022 [24]	7	20	35.00 (15.39–59.22)	24.71
Robinson et al. 2018 [23]	4	13	30.77 (9.09–61.43)	16.47
Tsai et al. 2014 [43]	1	7	14.29 (0.36–57.87)	9.41
Phan et al. 2006 [39]	0	3	0.00 (0.00–70.76)	4.71
Picca et al. 2001 [26]	1	2	50.00 (1.26–98.74)	3.53
Rajpoot and Gargus 2004 [40]	0	4	0.00 (0.00–60.24)	5.88
Total (fixed effects)	27	78	34.14 (24.19–45.23)	100

The mortality was observed to be higher among those with greater plasma ammonia concentration before CKRT. The pooled proportion (95% CI) of mortality among those with mean/median plasma ammonia ≥ 1000 µmol/L before CKRT was 31.94% (20.02–45.89) [I^2^: 21.11% (0.00–89.81); *p* = 0.2836; 4 studies, fixed-effects method; *n* = 51], and for < 1000 µmol/L before CKRT was 15.04% (6.65–27.67) [I^2^: 0.00% (0.00–0.00); *p* = 0.3355; 2 studies, fixed-effects method; *n* = 50].

On comparison across the different dialysis modalities, the pooled odds ratio (95% CI) of mortality was not significantly different between those who received CKRT vs. PD [0.664 (0.299–1.474) [I^2^: 7.97% (0.00–77.32); *p* = 0.3653; 6 studies, fixed-effects method]] and between those who received CKRT vs. HD [0.382 (0.129–1.127) [I^2^: 0.00% (0.00–96.23); *p* = 0.4109; 3 studies, fixed-effects method]]. The Egger’s test (*p* > 0.05) for all the above-pooled analyses indicates the absence of publication bias.

### Sensitivity analysis

The I^2^ value for sensitivity analyses for all the outcomes was similar to that obtained without excluding the studies based on the previously mentioned parameters (Tables 4 and 5). Also, the pooled results obtained from the sensitivity analyses were within the 95% CI of the overall pooled outcomes for all the parameters, indicating that the results of this meta-analysis are robust enough.

**Table 4:** Sensitivity analyses for time to 50% reduction in ammonia concentration based on the different parameters

Parameters	Dialysis	Number of studies	Sample size	Pooled estimate (95% CI)	I^2^ (95% CI), *p* value	Egger’s test (*p* value)
Neonates	CKRT	6	71	6.6 (5.0–8.2) hr	83.44% (65.34–92.09), *p* < 0.0001	0.8929
	PD	6	84	14.4 (13.2–15.5) hr	29.32% (0.00–71.07), *p* = 0.2152	0.1933
Non-neonates	CKRT	2	16	6.2 (2.7–9.7) hr	94.07% (81.22–98.12), *p* < 0.0001	< 0.0001
	PD	1	7	-	-	-
After 2015	CKRT	3	35	7.9 (5.9–10.0) hr	81.66% (43.13–94.08), *p* = 0.0043	0.7719
	PD	3	66	14.1 (12.9–15.3) hr	0.00% (0.00–3.9.45), *p* = 0.9461	0.1962
Before or during 2015	CKRT	5	52	5.5 (3.9–7.1) hr	83.2% (61.78–92.61), *p* = 0.0001	0.5635
	PD	4	25	17.5 (13.8–21.2) hr	28.46% (0.00–73.54), *p* = 0.2413	0.9169
Sample size ≥ 10	CKRT	5	66	7.2 (5.6–8.7) hr	86.39% (70.35–93.75), *p* < 0.0001	0.7756
	PD	3	66	14.1 (12.9 – 15.3) hr	0.00% (0.00–3.9.45), *p* = 0.9461	0.1962
Sample size < 10	CKRT	3	21	4.7 (3.7–5.7) hr	27.07% (0.00–97.55), *p* = 0.2538	0.5003
	PD	4	25	17.5 (13.8–21.2) hr	28.46% (0.00–73.54), *p* = 0.2413	0.9169

**Table 5:** Sensitivity analyses for mortality based on the different parameters

Parameters	Dialysis	Number of studies	Sample size	Pooled mortality (95% CI)	I^2^ (95% CI), *p* value	Egger’s test (*p* value)
Neonates	CKRT	16	288	27.71 (22.75–33.10)	33.27% (0.00–63.40), *p* = 0.0959	0.5918
	PD	11	115	34.77 (26.50–43.76)	21.80% (0.00–60.78), *p* = 0.2358	0.9391
	HD	5	68	37.95 (26.84–50.07)	27.13% (0.00–71.22), *p* = 0.2407	0.3492
Non-neonates	CKRT	9	142	18.87 (9.99–29.78)	56.71% (8.92–79.42), *p* = 0.0179	0.8317
	PD	1	1	-	-	-
	HD	2	10	13.91 (1.26–45.28)	0.00% (0.00–0.00), *p* = 0.5551	< 0.0001
After 2015	CKRT	17	359	21.84 (15.04–29.52)	56.96% (26.07–74.94), *p* = 0.0020	0.2010
	PD	5	57	37.61 (25.62–50.82)	47.91% (0.00–80.92), *p* = 0.1040	0.4874
	HD	3	62	40.65 (28.63–53.55)	0.00% (0.00–95.14), *p* = 0.5012	0.2421
During 2008–2015	CKRT	2	31	23.831 (5.915–48.892)	58.06% (0.00–90.05), *p* = 0.1226	< 0.0001
	PD	5	53	32.88 (21.11–46.46)	10.99% (0.00–82.57), *p* = 0.3433	0.0218
	HD	1	7	-	-	-
Before 2008	CKRT	6	40	29.04 (16.61–44.29)	0.00% (0.00–74.41), *p* = 0.4388	0.1879
	PD	2	6	21.87 (2.201–62.18)	0.00% (0.00–0.00), *p* = 0.7589	< 0.0001
	HD	3	9	13.77 (1.22–45.12)	24.09% (0.00–97.45), *p* = 0.2678	0.2559
Sample size ≥ 10	CKRT	16	381	21.02 (14.64–28.21)	57.61% (26.11–75.68), *p* = 0.0231	0.1288
	PD	4	76	33.249 (23.10–44.67)	23.97% (0.00–90.18), *p* = 0.3585	0.6418
	HD	3	62	40.65 (28.63–53.55)	0.00% (0.00–95.14), *p* = 0.1354	0.2421
Sample size < 10	CKRT	9	49	32.98 (21.20–46.57)	0.00% (0.00–61.80); *p* = 0.5027	0.0365
	PD	8	40	36.38 (22.98–51.52)	23.56% (0.00–65.00); *p* = 0.2415	0.1910
	HD	4	16	15.53 (3.45–38.52)	0.00% (0.00–85.70); *p* = 4387	0.6972

## Discussion

Intoxication-type IEMs resulting from aberrations in intermediary metabolic pathways often cause acute metabolic decompensation due to the accumulation of toxic compounds in conditions like UCD and OA [47] In these disorders, the onset of hyperammonemia occurs in the neonatal period, infanthood, and childhood in 40%, 30%, and 20% of cases, respectively [48]. In this systematic review, there was a greater cohort of neonates compared to pediatric patients. This is likely due to most IEM disorders causing hyperammonemia and requiring KRT presenting in the neonatal period. Current clinical guidelines for hyperammonemia management include limiting protein consumption and caloric intake ≥ 100 kcal/kg and pharmacological interventions (insulin, L-arginine, L-carnitine, vitamins, nitrogen scavengers (benzoate and/or phenylbutyrate), and peroral carboxyglutamate) [4]. MSUD is less commonly encountered in the neonatal population, with a prevalence rate of approximately 1:150,000 newborns [47]. Treatment for MSUD involves restricting protein intake and dietary leucine, providing high calories from carbohydrates and fat, and supplementing valine and isoleucine [47].

In neonates and children with symptomatic hyperammonemia or MSUD, the threshold to initiate KRT is variable. KRT should be initiated primarily through clinical decisions on the progressing medical status of the patient [4]. Generally, KRTs are considered when ammonia levels exceed 500 μmol/L or when there is no response within four hours after starting medical management for hyperammonemic patients [9]. KRT should also be considered in the presence of lower serum ammonia levels (300–500 umol/L) along with clinical indications of moderate to severe encephalopathy, coma, or seizure [4, 26]. Currently, there is no consensus on the appropriate timing to initiate dialysis for patients with elevated serum leucine levels.

A key strength of this systematic review is that it is the first to comprehensively analyze the efficacy and mortality outcomes of different modes of KRT in children and neonates with IEM and associated hyperammonemia. Findings from this review indicate that when comparing KRT modalities, CKRT demonstrated superior efficacy in mean percentage reduction in ammonia. When comparing time to a 50% reduction in ammonia levels, there was a trend that suggested HD was the fastest modality, while CKRT appeared superior to PD. All three modalities effectively reduced plasma leucine concentrations, with HD and CKRT showing the most substantial and consistent reductions. The findings of this study show that mortality showed a lower trend among neonates with IEMs who received CKRT vs. PD or HD, with a pooled proportion of mortality CKRT = 24.84% (*p* = 0.0061), PD = 34.42% (*p* = 0.2784), and HD = 34.14% (*p* = 0.1678).

IHD utilizes high dialysate flows for short periods. High flow rates make IHD optimal for rapid ammonium clearance; however, patients are often complicated with hypotension and transiently increased ammonia levels after discontinuation [4]. Consistent with our findings, IHD has been observed to have a faster ammonia reduction rate and requires less time to reach a 50% reduction in ammonia levels compared to other KRT modalities [14]. A study of 8 infants with hyperammonemia found the time for a 50% reduction in ammonia levels was 1–2 h in HD compared to 2–14 h in CVVHD, which were similar to results of 1.7 h (HD) and 2–14.5 h (CVVHD) [25]. Although the speed of ammonia reduction is superior in HD, it is associated with rebound hyperammonemia and hemodynamic instability. Additionally, the discontinuous nature of solute filtration results in inferior uremic and acid–base control [49]. Complications for IHD may be addressed using vasoactive medications or switching to CKRT or other hybrid therapy modalities to mitigate rebound hyperammonemia. A retrospective study investigating outcomes of KRT treatments of neonatal and pediatric IEM patients showed that 50% of the initial HD treatments required conversion to CVVHD for more precise control of metabolic disturbances [6].

CKRT is usually initiated with a low blood flow rate and gradually increases. CKRT decreases serum ammonia with a reduced risk of hemodynamic instability or ammonia rebound [25]. CKRT provides a distinct advantage compared to other modalities through continuous control of fluid status, control of acid–base status, ability to provide protein-rich nutrition while achieving uremic control, lower risk of infection, and a lower risk of cardiovascular complications. Disadvantages of CKRT include its complexity, requirement for anticoagulation, utilization of high fluid volumes, and higher cost compared to HD [49]. However, CKRT procedures for hyperammonemic children are often better tolerated than PD or IHD, and, when initiated promptly, result in improved long-term outcomes [26]. Discontinuation of CKRT in hyperammonemic patients is often guided by local practice, specifically focusing on the normalization of ammonia levels and the patient’s clinical status. Observationally, CKRT achieved the greatest efficacy of ammonia reduction, and the lowest mortality while HD achieved the best speed of ammonia reduction in IEM patients requiring KRT. For both mortality and efficacy outcomes, there was a trend towards PD displaying the poorest results.

PD involves the infusion of dialysate solution into the peritoneal cavity, which remains for a set period of time. This modality is distinctly suitable for children, rather than adults, due to the greater peritoneal surface area, which allows for more efficient solute clearance [50]. However, disadvantages associated with PD include the risk of peritonitis, variable shifts in serum glucose, fluid leaks, protein wasting, interference with diaphragm function, and residual uremia. PD is of limited efficacy in neonates with hyperammonemia when compared to other forms of KRT. This is supported by a 2010 study which concluded similarly that time to 50% reduction in ammonia levels and mortality outcomes were superior for patients receiving CVVHD compared to PD [29]. Other studies also concluded that PD is associated with a slower reduction in ammonia levels, increased mechanical complications, and difficulty obtaining precise fluid balance [13, 31]. Currently, PD largely remains an alternative option in the absence of other extracorporeal therapy due to facility resources or costs, and in neonates requiring timely vascular access where transport to another facility is not viable.

### Limitations

Limitations of this review include the exclusion of all efficacy outcomes from meta-analysis due to significant heterogeneity in published literature, particularly in the reported statistics for reduction rates and dialysis prescriptions. Furthermore, the availability of data on leucine levels for analysis was limited, suggesting potential areas for further research. While mortality was analyzed across each KRT modality, between-group comparisons should be taken cautiously as statistical significance was not tested.

## Conclusions

Due to the limitations in sample size in studies, it is difficult to directly compare CKRT, HD, and PD. However, there were trends noted in the analysis. CKRT showed a trend toward lower mortality rates compared to HD or PD, with trends suggesting earlier initiation of CKRT may be beneficial in lowering mortality. In terms of efficiency, there was a trend toward HD having a lower time to achieve 50% RIA, compared to CKRT, which trended towards having a lower time than PD. In terms of total % reduction of ammonia and leucine, all three modalities performed similarly. Given the variability in patient response and the complexity of IEMs, individualized treatment plans should be tailored to each patient's specific metabolic profile, with future research needed on between group comparisons of different KRT methods.

## Supplementary information

Below is the link to the electronic supplementary material.

Graphical abstract (PPTX 77 kb)

Supplementary file1 (DOCX 182 kb)

## Data Availability

All data generated or analyzed during this study are included in respective cited articles and their supplementary information files.

## Abbreviations

IEM: Inborn errors of metabolism
CKRT: Continuous kidney replacement therapy
CVVHD: Continuous venovenous hemodialysis
CVVH: Continuous venovenous hemofiltration
CVVHDF: Continuous venovenous hemodiafiltration
CAVH: Intermittent hemodialysis, peritoneal dialysis, continuous arteriovenous hemofiltration
CAVHD: Continuous arteriovenous hemodialysis
CAVHDF: Continuous arteriovenous hemodiafiltration
