# Supplementary material for: Inborn errors of metabolism in neonates and pediatrics on varying dialysis modalities: a systematic review and meta-analysis
Source: Pediatr Nephrol. 2024 Nov 11;40(7):2177–88. doi: 10.1007/s00467-024-06547-7 (PMC12116838; doi:10.1007/s00467-024-06547-7)
Supplement: Supplementary file 1 — Graphical abstract (PPTX 77 kb) [file 467_2024_6547_MOESM1_ESM.pptx]

## Slide 1
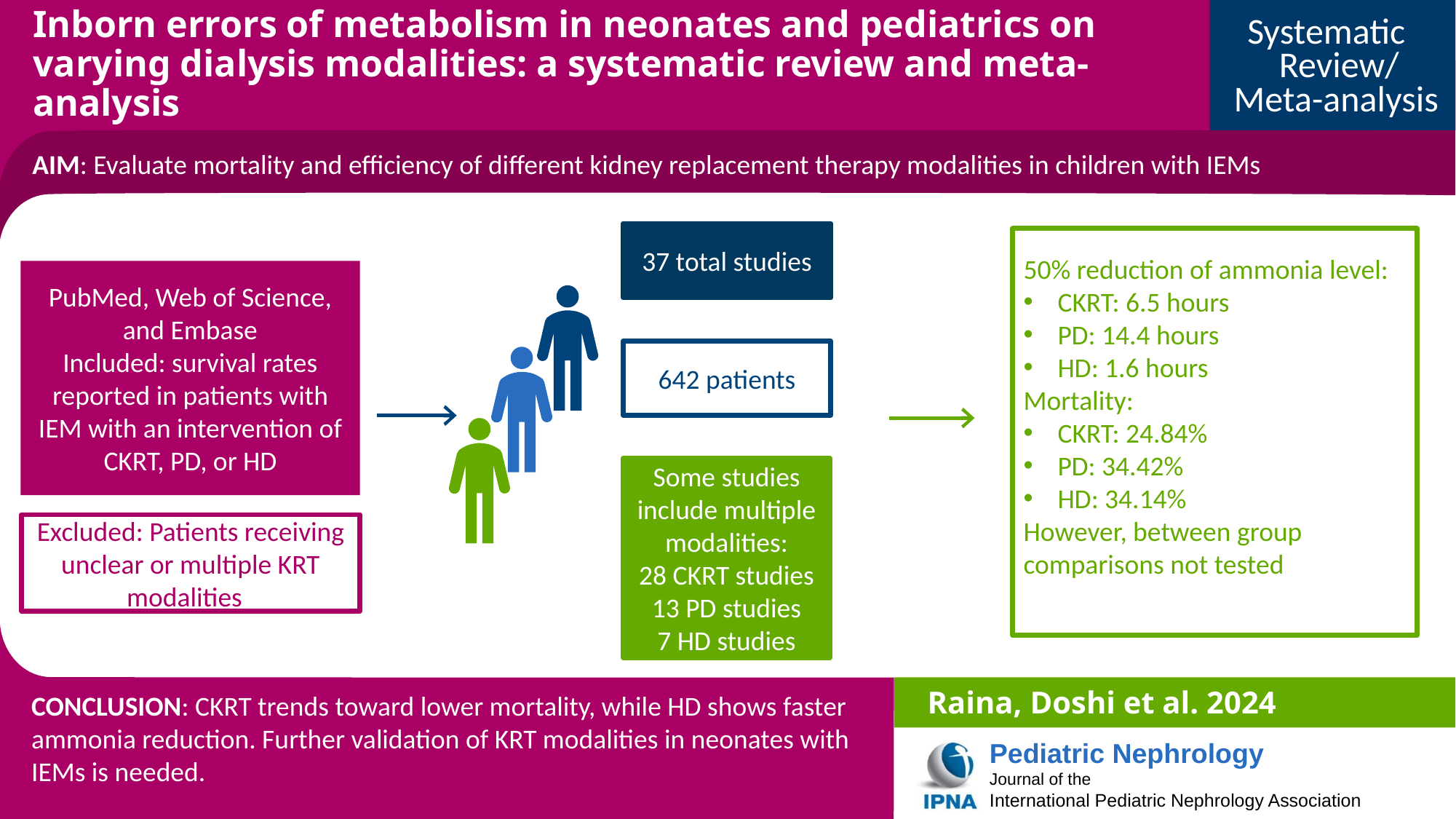

Inborn errors of metabolism in neonates and pediatrics on varying dialysis modalities: a systematic review and meta-analysis
AIM: Evaluate mortality and efficiency of different kidney replacement therapy modalities in children with IEMs
37 total studies
50% reduction of ammonia level:
CKRT: 6.5 hours
PD: 14.4 hours
HD: 1.6 hours
Mortality:
CKRT: 24.84%
PD: 34.42%
HD: 34.14%
However, between group comparisons not tested
PubMed, Web of Science, and Embase
Included: survival rates reported in patients with IEM with an intervention of CKRT, PD, or HD
642 patients
Some studies include multiple modalities:
28 CKRT studies
13 PD studies
7 HD studies
Excluded: Patients receiving unclear or multiple KRT modalities
Raina, Doshi et al. 2024
CONCLUSION: CKRT trends toward lower mortality, while HD shows faster ammonia reduction. Further validation of KRT modalities in neonates with IEMs is needed.
