# Supplementary material for: Inborn errors of metabolism in neonates and pediatrics on varying dialysis modalities: a systematic review and meta-analysis
Source: Pediatr Nephrol. 2024 Nov 11;40(7):2177–88. doi: 10.1007/s00467-024-06547-7 (PMC12116838; doi:10.1007/s00467-024-06547-7)
Supplement: Supplementary file 2 — Supplementary file1 (DOCX 182 kb) [file 467_2024_6547_MOESM2_ESM.docx]

**Supplemental Data Appendix:**

**Table S1: Detailed Search Strategy**

1. **Continuous Kidney Replacement Therapy (CKRT)**

| Database | PubMed | EMBASE | Web of Science |
| --- | --- | --- | --- |
| Date | 1-15-2023 | 1-15-2023 | 1-15-2023 |
| Strategy 1. | “Inborn errors of Metabolism” [Mesh Terms] | “Inborn errors of Metabolism” [Title, Abstract, Author Keywords] | “Inborn errors of Metabolism” [ALL] |
| 2. | “Urea Cycle Disorders” [Title/Abstract] | “IEM” [All fields] | “Hyperammonemia” [ALL] |
| 3. | “Hyperammonemia” [Title/Abstract] | “Urea Cycle Disorders” [Title, Abstract, Author Keywords] | “Urea Cycle Disorders” [ALL] |
| 4. | 1 or 2 or 3 | “Hyperammonemia” [Title, Abstract, Author Keywords] | 1 or 2 or 3 |
| 5. | “Neonates” [Mesh Terms] | 1 or 2 or 3 or 4 | “Neonates” [ALL] |
| 6. | “Children” [Title/Abstract] | “Neonates” [Title, Abstract, Author Keywords] | “Pediatrics” [ALL] |
| 7. | “Pediatrics” [Title/Abstreact] | “Children” [Title, Abstract, Author Keywords] | 5 or 6 |
| 8. | 5 or 6 or 7 | “Pediatrics” [Title, Abstract, Author Keywords] | “Continuous Renal Replacement Therapy” [ALL] |
| 9. | “Continuous Renal Replacement Therapy” [Title/Abstract] | 6 or 7 or 8 | 4 and 7 and 8 |
| 10. | 4 and 8 and 9 | “Continuous Renal Replacement Therapy” [Title, Abstract, Author Keywords] | Limit 9 to English |
| 11. | Limit 10 to English | 5 and 9 and 10 |  |
| 12. |  | Limit 11 to English |  |
| Results | 74 | 211 | 123 |

**b) Peritoneal Dialysis**

| Database | PubMed | EMBASE | Web of Science |
| --- | --- | --- | --- |
| Date | 1-15-2023 | 1-15-2023 | 1-15-2023 |
| Strategy 1. | “Inborn errors of Metabolism” [Mesh Terms] | “Inborn errors of Metabolism” [Title, Abstract, Author Keywords] | “Inborn errors of Metabolism” [ALL] |
| 2. | “Urea Cycle Disorders” [Title/Abstract] | “IEM” [All fields] | “Hyperammonemia” [ALL] |
| 3. | “Hyperammonemia” [Title/Abstract] | “Urea Cycle Disorders” [Title, Abstract, Author Keywords] | “Urea Cycle Disorders” [ALL} |
| 4. | 1 or 2 or 3 | “Hyperammonemia” [Title, Abstract, Author Keywords] | 1 or 2 or 3 |
| 5. | “Neonates” [Mesh Terms] | 1 or 2 or 3 or 4 | “Neonates” [ALL] |
| 6. | “Children” [Title/Abstract] | “Neonates” [Title, Abstract, Author Keywords] | “Pediatrics” [ALL] |
| 7. | “Pediatrics” [Title/Abstreact] | “Children” [Title, Abstract, Author Keywords] | 5 or 6 |
| 8. | 5 or 6 or 7 | “Pediatrics” [Title, Abstract, Author Keywords] | “Peritoneal Dialysis” [ALL] |
| 9. | “Peritoneal Dialysis” [Title/Abstract] | 6 or 7 or 8 | 4 and 7 and 8 |
| 10. | 4 and 8 and 9 | “Peritoneal Dialysis” [Title, Abstract, Author Keywords] | Limit 9 to English |
| 11. | Limit 10 to English | 5 and 9 and 10 |  |
| 12. |  | Limit 11 to English |  |
| Results | 97 | 89 | 88 |

**c) Hemodialysis**

| Database | PubMed | EMBASE | Web of Science |
| --- | --- | --- | --- |
|  |  |  |  |
| Date | 1-15-2023 | 1-15-2023 | 1-15-2023 |
| Strategy 1. | “Inborn errors of Metabolism” [Mesh Terms] | “Inborn errors of Metabolism” [Title, Abstract, Author Keywords] | “Inborn errors of Metabolism” [ALL] |
| 2. | “Urea Cycle Disorders” [Title/Abstract] | “IEM” [All fields] | “Hyperammonemia” [ALL] |
| 3. | “Hyperammonemia” [Title/Abstract] | “Urea Cycle Disorders” [Title, Abstract, Author Keywords] | “Urea Cycle Disorders” [ALL} |
| 4. | 1 or 2 or 3 | “Hyperammonemia” [Title, Abstract, Author Keywords] | 1 or 2 or 3 |
| 5. | “Neonates” [Mesh Terms] | 1 or 2 or 3 or 4 | “Neonates” [ALL] |
| 6. | “Children” [Title/Abstract] | “Neonates” [Title, Abstract, Author Keywords] | “Pediatrics” [ALL] |
| 7. | “Pediatrics” [Title/Abstreact] | “Children” [Title, Abstract, Author Keywords] | 5 or 6 |
| 8. | 5 or 6 or 7 | “Pediatrics” [Title, Abstract, Author Keywords] | “Hemodialysis” [ALL] |
| 9. | “Hemodialysis” [Title/Abstract] | 6 or 7 or 8 | 4 and 7 and 8 |
| 10. | 4 and 8 and 9 | “Hemodialysis” [Title, Abstract, Author Keywords] | Limit 9 to English |
| 11. | Limit 10 to English | 5 and 9 and 10 |  |
| 12. |  | Limit 11 to English |  |
| Results | 164 | 205 | 75 |

**Table S2: PICOS Inclusion/Exclusion Criteria**

| Criteria | Inclusion Criteria | Exclusion Criteria |
| --- | --- | --- |
| Population | > 1 patients with Inborn Errors of Metabolism, Urea Cycle Disorders (UCD), Organic Acidemia (OA), Hyperammonemia, Neonates (age of 4 weeks or less), Pediatrics (<18 years of age) | Patients without a diagnosis of Inborn Errors of Metabolism, UCD, or Hyperammonemia  Patients >18 years of age |
| Intervention | Continuous Kidney Replacement Therapy (CKRT), Continuous veno-venous hemodialysis (CVVHD), continuous veno-venous hemofiltration (CVVH), continuous veno-venous hemodiafiltration (CVVHDF) | Patients with IEM, UCD, Hyperammonemia not on KRT that is continuous (CKRT, CVVHD, CVVH, CVVHDF) |
| Comparison | Peritoneal Dialysis (PD), Hemodialysis (HD) | Patients with IEM not on KRT or PD or HD |
| Outcomes | Mortality  Efficacy- % reduction in ammonia / leucine concentration from pre-dialysis to post-dialysis and time to 50% reduction in ammonia / leucine level | No follow-up reported, non-efficacy or mortality outcomes (secondary complications, extraorgan outcomes (cardiovascular, kidney, pulmonary, neurological, etc.) |
| Study Types | Cross sectional studies, prospective and retrospective studies, randomized clinical trial, control studies and observational studies, case reports | Systematic Reviews, Literature Reviews |

**Table S3: Description of Included Studies**

| **Title** | | | | **Author** | | |  | | |  | | | Age (at admission or at dialysis) | | | | | | | |  | | | | **Total Sample Size** | | | **CRRT** | | | | | | | | | | | | | | | | | | | | | | | | | | | | | | | | | | | | | | | | | | | | | | | | | | **PD** | | | | | | | | | | | | | | | | | | | | | | | | | | | | | | | | | | | | | | | | | | | | | | | | | | | | | | | | | | | | | | | | | | | | | | | | | | | | | | | | | | | | | | | | | | **HD** | | | | | | | | | | | | | | | | | | | | | | | | | | | | | | | | | | | | | | | | | | | | | | | | | | | | | | | | | |  |  |  |  |  |
| --- | --- | --- | --- | --- | --- | --- | --- | --- | --- | --- | --- | --- | --- | --- | --- | --- | --- | --- | --- | --- | --- | --- | --- | --- | --- | --- | --- | --- | --- | --- | --- | --- | --- | --- | --- | --- | --- | --- | --- | --- | --- | --- | --- | --- | --- | --- | --- | --- | --- | --- | --- | --- | --- | --- | --- | --- | --- | --- | --- | --- | --- | --- | --- | --- | --- | --- | --- | --- | --- | --- | --- | --- | --- | --- | --- | --- | --- | --- | --- | --- | --- | --- | --- | --- | --- | --- | --- | --- | --- | --- | --- | --- | --- | --- | --- | --- | --- | --- | --- | --- | --- | --- | --- | --- | --- | --- | --- | --- | --- | --- | --- | --- | --- | --- | --- | --- | --- | --- | --- | --- | --- | --- | --- | --- | --- | --- | --- | --- | --- | --- | --- | --- | --- | --- | --- | --- | --- | --- | --- | --- | --- | --- | --- | --- | --- | --- | --- | --- | --- | --- | --- | --- | --- | --- | --- | --- | --- | --- | --- | --- | --- | --- | --- | --- | --- | --- | --- | --- | --- | --- | --- | --- | --- | --- | --- | --- | --- | --- | --- | --- | --- | --- | --- | --- | --- | --- | --- | --- | --- | --- | --- | --- | --- | --- | --- | --- | --- | --- | --- | --- | --- | --- | --- | --- | --- | --- | --- | --- | --- | --- | --- | --- | --- | --- | --- | --- | --- | --- | --- | --- | --- | --- | --- | --- | --- | --- | --- | --- | --- | --- |
|  |  |  |  |  |  |  | **Publication Year** | | | **Country** | | | **age at admission (days); reported as  median [IQR] (range) *mean ± SD (range)** | | | | **age at dialysis (days); reported as  median [IQR] (range) *mean ± SD (range)** | | | | **Time to RRT initiation from symptom onset  (h), mean±SD (min–max), *=from admission** | | | |  |  |  | n | | | **Duration of dialysis (hours);median [IQR] (range) *mean ± SD (range)** | | | | **Pre-CRRT Ammonia level  (µmol/L), median, (Range),  *=mean ± SD** | | | | **Post-CKRT Ammonia level  (µmol/L), median (Range) *=mean ± SD** | | | | | **ammonia reduction in 24 hours; reported as  median [IQR] (range) *mean ± SD (range)** | | | | **Time to ammonia <200 µmol/l (hours)** | | | **Pre-CKRT Leucine level  (µmol/L), Median (Range),  *=mean ± SD** | | | | **Post-CKRT Leucine level  (µmol/L), Median (Range),  *=mean ± SD** | | | **Leucine reduction rate (% per hour) *=mean ± SD** | | | | **Median time to reach a 50% decrease of the initial ammonia level (hours)  *=Mean, (Range)** | | | **Median time to reach a 50% decrease of the initial leucine level (hours)  *=Mean, (Range)** | | | | | | | **Died [n]** | | | | | | **n** | | | | | **duration of dialysis (hours); reported as  median [IQR] (range) *mean ± SD (range)** | | | | | | | **Pre-PD Ammonia level  (µmol/L); reported as  median [IQR] (range) *mean ± SD (range)** | | | | | | **Post-PD Ammonia level  (µmol/L), median (Range) *=mean ± SD** | | | | | | | **ammonia reduction in 24 hours (%); reported as  median [IQR] (range) *mean ± SD (range)** | | | | | | | | **Time to ammonia <200 µmol/l (hours); reported as  median [IQR] (range) *mean ± SD (range)** | | | | | | | | **Ammonia reduction rate (% per hour); reported as  median [IQR] (range) *mean ± SD (range)** | | | | | | **time to reach a 50% decrease of the initial ammonia level (hours); reported as  median [IQR] (range) *mean ± SD (range)** | | | | | | | | | **Pre-PD Leucine level  (µmol/L), ; reported as  median [IQR] (range) *mean ± SD (range)** | | | | | | | | **Post-PD Leucine level  (µmol/L),; reported as  median [IQR] (range) *mean ± SD (range)** | | | | | | | | **Leucine reduction rate (% per hour); reported as  median [IQR] (range) *mean ± SD (range)** | | | | | | **time to reach a 50% decrease of the initial leucine level (hours); reported as  median [IQR] (range) *mean ± SD (range)** | | | | | | | **Died [n]** | | | | | | | | **n** | | | | **duration of dialysis (hours); reported as  median [IQR] (range) *mean ± SD (range)** | | | | | **Pre-HD Ammonia level  (µmol/L); reported as  median [IQR] (range) *mean ± SD (range)** | | | | **Post-HD Ammonia level  (µmol/L), median (Range) *=mean ± SD** | | | | **ammonia reduction in 24 hours (%); reported as  median [IQR] (range) *mean ± SD (range)** | | | | **Time to ammonia <200 µmol/l (hours); reported as  median [IQR] (range) *mean ± SD (range)** | | | **Time to normalization (hours); reported as  median [IQR] (range) *mean ± SD (range)** | | | | | | **time to reach a 50% decrease of the initial ammonia level (hours); reported as  median [IQR] (range) *mean ± SD (range)** | | | | | **Pre-PD Leucine level  (µmol/L), ; reported as  median [IQR] (range) *mean ± SD (range)** | | | | **Post-PD Leucine level  (µmol/L),; reported as  median [IQR] (range) *mean ± SD (range)** | | | | | **Leucine reduction rate (% per hour); reported as  median [IQR] (range) *mean ± SD (range)** | | | | | **time to reach a 50% decrease of the initial leucine level (hours); reported as  median [IQR] (range) *mean ± SD (range)** | | | | **Died [n]** | | | | | |  |
| Characteristics of continuous venovenous hemodiafiltration in the acute treatment of inherited metabolic disorders[10] | | | | Eminoğlu FT et al. | | | 2022 | | | Turkey | | |  | | | | 72.3 [9.9–1040.8] | | | | *89.3±144.3 (6–720) | | | | 22 | | | 22 | | | *32.6 ± 22.2 (11–48) | | | | *1279.9 ± 612.1(451–2626) | | | | *85.1 ± 21.6 (56–140) | | | | |  | | | |  | | | *1608.4±885.3 (733–3330) | | | | *314.6±109.9 (139–580) | | |  | | | |  | | |  | | | | | | | 8 | | | | | |  | | | | | | | | | | | | | | | | | | | | | | | | | | | | | | | | | | | | | | | | | | | | | | | | | | | | | | | | | | | | | | | | | | | | | | | | | | | | | | | | | | | | | | | | | | | | |  | | | | | | | | | | | | | | | | | | | | | | | | | | | | | | | | | | | | | | | | | | | | | | | | | | | | | | | | | | |  |
| Continuous venovenous hemodiafiltration in the treatment of newborns with an inborn metabolic disease: a single center experience[11] | | | | Akduman H et al. | | | 2020 | | | Turkey | | | *10 ± 8.6 (3–28) | | | |  | | | |  | | | | 8 | | | 8 | | | *32.3 ± 11.1 (16-44) | | | | *1097 (451–1728) | | | | *190.5 (96–484) | | | | |  | | | |  | | | *2053.5 ± 1282 | | | | *473.5 ± 7.8 | | |  | | | |  | | |  | | | | | | | 4 | | | | | |  |  |  |  |  |  |  |  |  |  |  |  |  |  |  |  |  |  |  |  |  |  |  |  |  |  |  |  |  |  |  |  |  |  |  |  |  |  |  |  |  |  |  |  |  |  |  |  |  |  |  |  |  |  |  |  |  |  |  |  |  |  |  |  |  |  |  |  |  |  |  |  |  |  |  |  |  |  |  |  |  |  |  |  |  |  |  |  |  |  |  |  |  |  |  |  |  |  |  |  |  |  |  |  |  |  |  |  |  |  |  |  |  |  |  |  |  |  |  |  |  |  |  |  |  |  |  |  |  |  |  |  |  |  |  |  |  |  |  |  |  |  |  |  |  |  |  |  |  |  |  |  |  |
| Management of 35 critically ill hyperammonemic neonates: Role of early administration of metabolite scavengers and continuous hemodialysis [12] | | | | Abily-Donval L et al. | | | 2020 | | | France | | | *4 (3–32) | | | |  | | | | 26.4 (4.8-96) | | | | 14 | | | 14 | | | 11 h (6–18) | | | | 644 (298–1247) | | | | 52 (25–150) | | | | |  | | | |  | | |  | | | |  | | |  | | | | 5 (3–12) | | |  | | | | | | | 3 | | | | | |  |  |  |  |  |  |  |  |  |  |  |  |  |  |  |  |  |  |  |  |  |  |  |  |  |  |  |  |  |  |  |  |  |  |  |  |  |  |  |  |  |  |  |  |  |  |  |  |  |  |  |  |  |  |  |  |  |  |  |  |  |  |  |  |  |  |  |  |  |  |  |  |  |  |  |  |  |  |  |  |  |  |  |  |  |  |  |  |  |  |  |  |  |  |  |  |  |  |  |  |  |  |  |  |  |  |  |  |  |  |  |  |  |  |  |  |  |  |  |  |  |  |  |  |  |  |  |  |  |  |  |  |  |  |  |  |  |  |  |  |  |  |  |  |  |  |  |  |  |  |  |  |  |
| Continuous Renal Replacement Therapy with High Flow Rate Can Effectively, Safely, and Quickly Reduce Plasma Ammonia and Leucine Levels in Children [13]. | | | | Aygun F et al. | | | 2019 | | | Turkey | | | *1376.1 ± 1719.2 | | | |  | | | |  | | | | 36 | | | 36 | | | *36.42 ± 81.17 | | | | MSUD:*164.8 ± 145.5, UCD: *1143.5 ± 1069.9, OA: *538.6 ± 1017.9 | | | | MSUD: *57.7 ± 51.4, UCD= *50.0 ± 7.07, OA: *71.23 ± 55.1 | | | | |  | | | |  | | | *1777.0 ± 831.6 | | | | *222.1 ± 143.5 | | | *3.88 ± 3.65 | | | |  | | |  | | | | | | | 4 | | | | | |  |  |  |  |  |  |  |  |  |  |  |  |  |  |  |  |  |  |  |  |  |  |  |  |  |  |  |  |  |  |  |  |  |  |  |  |  |  |  |  |  |  |  |  |  |  |  |  |  |  |  |  |  |  |  |  |  |  |  |  |  |  |  |  |  |  |  |  |  |  |  |  |  |  |  |  |  |  |  |  |  |  |  |  |  |  |  |  |  |  |  |  |  |  |  |  |  |  |  |  |  |  |  |  |  |  |  |  |  |  |  |  |  |  |  |  |  |  |  |  |  |  |  |  |  |  |  |  |  |  |  |  |  |  |  |  |  |  |  |  |  |  |  |  |  |  |  |  |  |  |  |  |  |
| Optimal Prescriptions of Continuous Renal Replacement Therapy in Neonates with Hyperammonemia [14]. | | | | Kim JY et al. | | | 2019 | | | South Korea | | |  | | | | 4 (3–9) | | | | 24 (24–120) | | | | 12 | | | 12 | | | 120 (48–192) | | | | 1,320 (373–3,307) | | | |  | | | | |  | | | |  | | |  | | | |  | | |  | | | | 12.8 | | |  | | | | | | | 2 | | | | | |  |  |  |  |  |  |  |  |  |  |  |  |  |  |  |  |  |  |  |  |  |  |  |  |  |  |  |  |  |  |  |  |  |  |  |  |  |  |  |  |  |  |  |  |  |  |  |  |  |  |  |  |  |  |  |  |  |  |  |  |  |  |  |  |  |  |  |  |  |  |  |  |  |  |  |  |  |  |  |  |  |  |  |  |  |  |  |  |  |  |  |  |  |  |  |  |  |  |  |  |  |  |  |  |  |  |  |  |  |  |  |  |  |  |  |  |  |  |  |  |  |  |  |  |  |  |  |  |  |  |  |  |  |  |  |  |  |  |  |  |  |  |  |  |  |  |  |  |  |  |  |  |  |
| The Role of Supportive Treatment in the Management of Hyperammonemia in Neonates and Infants [15] | | | | Demirkol D et al. | | | 2019 | | | Turkey | | | 5 (2-150) | | | |  | | | |  | | | | 10 | | | 10 | | | 40 (24-89) | | | | 2,132 (551–4,367) | | | | 135 | | | | |  | | | |  | | |  | | | |  | | |  | | | | 8 (3–15 ) | | |  | | | | | | | 1 | | | | | |  |  |  |  |  |  |  |  |  |  |  |  |  |  |  |  |  |  |  |  |  |  |  |  |  |  |  |  |  |  |  |  |  |  |  |  |  |  |  |  |  |  |  |  |  |  |  |  |  |  |  |  |  |  |  |  |  |  |  |  |  |  |  |  |  |  |  |  |  |  |  |  |  |  |  |  |  |  |  |  |  |  |  |  |  |  |  |  |  |  |  |  |  |  |  |  |  |  |  |  |  |  |  |  |  |  |  |  |  |  |  |  |  |  |  |  |  |  |  |  |  |  |  |  |  |  |  |  |  |  |  |  |  |  |  |  |  |  |  |  |  |  |  |  |  |  |  |  |  |  |  |  |  |
| Renal replacement therapy in the neonatal intensive care unit [16]. | | | | Mok TYD et al. | | | 2018 | | | Taiwan | | |  | | | | 4 (2-30) | | | |  | | | | 3 | | | 3 | | | 72 (24-72 ) | | | |  | | | |  | | | | | 87.20% | | | |  | | |  | | | |  | | |  | | | |  | | |  | | | | | | | 0 | | | | | |  |  |  |  |  |  |  |  |  |  |  |  |  |  |  |  |  |  |  |  |  |  |  |  |  |  |  |  |  |  |  |  |  |  |  |  |  |  |  |  |  |  |  |  |  |  |  |  |  |  |  |  |  |  |  |  |  |  |  |  |  |  |  |  |  |  |  |  |  |  |  |  |  |  |  |  |  |  |  |  |  |  |  |  |  |  |  |  |  |  |  |  |  |  |  |  |  |  |  |  |  |  |  |  |  |  |  |  |  |  |  |  |  |  |  |  |  |  |  |  |  |  |  |  |  |  |  |  |  |  |  |  |  |  |  |  |  |  |  |  |  |  |  |  |  |  |  |  |  |  |  |  |  |
| Continuous Venovenous Hemodiafiltration in the Treatment of Maple Syrup Urine Disease [17] | | | | Demirkol D et al. | | | 2016 | | | Turkey | | | *1241 ± 955 (15-2646.3) | | | |  | | | |  | | | | 14 | | | 14 | | | *20.2 ± 8.6 (9–36) | | | |  | | | |  | | | | |  | | | |  | | | *1,648 ± 623.8 (714-2,768) | | | | *256.5 ± 150.6 (117-646) | | |  | | | |  | | |  | | | | | | | 0 | | | | | |  |  |  |  |  |  |  |  |  |  |  |  |  |  |  |  |  |  |  |  |  |  |  |  |  |  |  |  |  |  |  |  |  |  |  |  |  |  |  |  |  |  |  |  |  |  |  |  |  |  |  |  |  |  |  |  |  |  |  |  |  |  |  |  |  |  |  |  |  |  |  |  |  |  |  |  |  |  |  |  |  |  |  |  |  |  |  |  |  |  |  |  |  |  |  |  |  |  |  |  |  |  |  |  |  |  |  |  |  |  |  |  |  |  |  |  |  |  |  |  |  |  |  |  |  |  |  |  |  |  |  |  |  |  |  |  |  |  |  |  |  |  |  |  |  |  |  |  |  |  |  |  |  |
| Continuous hemofiltration in the control of neonatal hyperammonemia: a 10-year experience [18] | | | | Westrope C et al. | | | 2010 | | | United Kingdom | | | 4 (1–10 ) | | | |  | | | |  | | | | 14 | | | 14 | | | *49 (6–94) | | | |  | | | |  | | | | | 87%, *81±3.5% | | | |  | | |  | | | |  | | |  | | | | 7.4 (4.5–14.3) | | |  | | | | | | | 5 | | | | | |  |  |  |  |  |  |  |  |  |  |  |  |  |  |  |  |  |  |  |  |  |  |  |  |  |  |  |  |  |  |  |  |  |  |  |  |  |  |  |  |  |  |  |  |  |  |  |  |  |  |  |  |  |  |  |  |  |  |  |  |  |  |  |  |  |  |  |  |  |  |  |  |  |  |  |  |  |  |  |  |  |  |  |  |  |  |  |  |  |  |  |  |  |  |  |  |  |  |  |  |  |  |  |  |  |  |  |  |  |  |  |  |  |  |  |  |  |  |  |  |  |  |  |  |  |  |  |  |  |  |  |  |  |  |  |  |  |  |  |  |  |  |  |  |  |  |  |  |  |  |  |  |  |
| Continuous venovenous hemodiafiltration in neonatal onset hyperammonemia [19] | | | | Hiroma T et al. | | | 2002 | | | Japan | | |  | | | |  | | | |  | | | | 4 | | | 4 | | |  | | | | *260 -1740 | | | |  | | | | |  | | | | 30 | | |  | | | |  | | |  | | | |  | | |  | | | | | | | 2 | | | | | |  |  |  |  |  |  |  |  |  |  |  |  |  |  |  |  |  |  |  |  |  |  |  |  |  |  |  |  |  |  |  |  |  |  |  |  |  |  |  |  |  |  |  |  |  |  |  |  |  |  |  |  |  |  |  |  |  |  |  |  |  |  |  |  |  |  |  |  |  |  |  |  |  |  |  |  |  |  |  |  |  |  |  |  |  |  |  |  |  |  |  |  |  |  |  |  |  |  |  |  |  |  |  |  |  |  |  |  |  |  |  |  |  |  |  |  |  |  |  |  |  |  |  |  |  |  |  |  |  |  |  |  |  |  |  |  |  |  |  |  |  |  |  |  |  |  |  |  |  |  |  |  |  |
| Efficacy of Peritoneal Dialysis in Neonates Presenting With Hyperammonaemia Due to Urea Cycle Defects and Organic Acidaemia [20] | | | | Celik M et al. | | | 2019 | | | Turkey | | | 3 [2-10.2] (2-28) | | | |  | | | |  | | | | 14 | | |  | | | | | | | | | | | | | | | | | | | | | | | | | | | | | | | | | | | | | | | | | | | | | | | | | | 14 |  | | | | | | | **Survivors:** 1652 [1165-2098] **Non-Survivors:** 1289 [1070-5550] | | | | | | | **Survivors:** 89 [69-103] **Non-Survivors:** 758 [112-1006] | | | | | | | | **Survivors:** 81.1 [75-92]  **Non-survivors:** 44.3 [-1.8-82.3] | | | | | | | **Survivors:** 19.5 [9.2-46.5] **Non-Survivors:** 96 [25.5-186] | | | | | | |  | | | | | | | | ***Survivors:** 16.1 ± 9.2  ***Non-survivors:** 12 ± 0 | | | | | | | |  | | | | | | |  | | | | | | | |  | | | | | | | |  | | | | | | | 6 | | | | | | | |  | | | | | | | | | | | | | | | | | | | | | | | | | | | | | | | | | | | | | | | | | | | | | | | | | | | | | | | | | | | | | |
| Short-term survival of hyperammonemic neonates treated with dialysis [21] | | | | Picca S et al. | | | 2015 | | | Italy | | |  | | | | 4.4 (3.0-12.0) | | | |  | | | | 23 | | |  |  |  |  |  |  |  |  |  |  |  |  |  |  |  |  |  |  |  |  |  |  |  |  |  |  |  |  |  |  |  |  |  |  |  |  |  |  |  |  |  |  |  |  |  |  |  |  |  |  | 23 | 52.8 (death-censored) | | | | | | | 980 (402–3,212) | | | | | | |  | | | | | | | |  | | | | | | |  | | | | | | | *14.8 ±11.0 | | | | | | | |  | | | | | | | |  | | | | | | |  | | | | | | | |  | | | | | | | |  | | | | | | | 4 | | | | | | | |  |  |  |  |  |  |  |  |  |  |  |  |  |  |  |  |  |  |  |  |  |  |  |  |  |  |  |  |  |  |  |  |  |  |  |  |  |  |  |  |  |  |  |  |  |  |  |  |  |  |  |  |  |  |  |  |  |  |  |  |  |  |
| Utility of peritoneal dialysis in neonates affected by inborn errors of metabolism [22] | | | | Bilgin L et al. | | | 2014 | | | Turkey | | | *11.7 ± 9.7 | | | |  | | | |  | | | | 9 | | |  |  |  |  |  |  |  |  |  |  |  |  |  |  |  |  |  |  |  |  |  |  |  |  |  |  |  |  |  |  |  |  |  |  |  |  |  |  |  |  |  |  |  |  |  |  |  |  |  |  | 9 | *110.4 ± 45.6 | | | | | | | *1003 ± 393 | | | | | | | *300 ± 440 | | | | | | | |  | | | | | | | *52.8 ± 26.2 | | | | | | |  | | | | | | | | 24 (8-36) | | | | | | | | *3117 ± 873 | | | | | | | *811 ± 141 | | | | | | | |  | | | | | | | | 24 (18-36) | | | | | | | 4 | | | | | | | |  |  |  |  |  |  |  |  |  |  |  |  |  |  |  |  |  |  |  |  |  |  |  |  |  |  |  |  |  |  |  |  |  |  |  |  |  |  |  |  |  |  |  |  |  |  |  |  |  |  |  |  |  |  |  |  |  |  |  |  |  |  |
| Rapid Resolution of Hyperammonemia in Neonates Using Extracorporeal Membrane Oxygenation as a Platform to Drive Hemodialysis [23] | | | Robinson JR et al. | | 2018 | | | United States | | | 3 [2–5] | | | | |  | | |  | | | 13 | | | |  | | | | | | | | | | | | | | | | | | | | | | | | | | | | | | | | | | | | | | | | | | |  | | | | | | | | | | | | | | | | | | | | | | | | | | | | | | | | | | | | | | | | | | | | | | | | | | | | | | | | | 13 | | | | | | | | | 50.3 [33.1-70.3] | | | | | | 998 [902-1476] | | | | | | | |  | | | | | | | |  | | | | | | | 4.5 [3.6 - 13.5] (<300 umol/L) | | | | | | | | | | 7.3 [3.6-13.5] | | | |  | | | | | | | | |  | | | | | | | 92.1 | | | | | | | |  | | | | | | | | |  | | | | | | | | 4 | | | | | | | |  |  |  |  |
| Acute hemodialysis therapy in neonates with inborn errors of metabolism [24] | | | Eisenstein I et al. | | 2022 | | | Germany | | | 5 [3–8] | | | | |  | | |  | | | 20 | | | |  |  |  |  |  |  |  |  |  |  |  |  |  |  |  |  |  |  |  |  |  |  |  |  |  |  |  |  |  |  |  |  |  |  |  |  |  |  |  |  |  |  |  |  |  |  |  |  |  |  |  |  |  |  |  |  |  |  |  |  |  |  |  |  |  |  |  |  |  |  |  |  |  |  |  |  |  |  |  |  |  |  |  |  |  |  |  |  |  |  |  |  |  |  |  |  |  |  |  |  | 20 | | | | | | | | |  | | | | | | *955 ± 444 (273-1670) | | | | | | | | 129 ± 55 | | | | | | | |  | | | | | | |  | | | | | | | | | |  | | | |  | | | | | | | | | *2281 ± 631 (1425 - 3320) | | | | | | | *179 ± 91 | | | | | | | |  | | | | | | | | |  | | | | | | | | 7 | | | | | | | |  |  |  |  |
| High-volume continuous venovenous hemofiltration as an effective therapy for acute management of inborn errors of metabolism in young children [25]. | | | Lai YC et al. | | 2007 | | | Taiwan | | |  | | | | | *730 (11-2555) | | |  | | | 6 | | | | 5 | | |  | | | |  | | | |  | | | |  | | | |  | | | |  | | | |  | | |  | | | | *6.63 (2–14.5) | | |  | | | | 1 | | | | 1 | | | | |  | | | |  | | | |  | | | | |  | |  | | | | | |  | | | 12 | | | | | |  | | | |  | | | |  | | | |  | | | 0 | | | | | | |  | | | | | | | | | | | | | | | | | | | | | | | | | | | | | | | | | | | | | | | | | | | | | | | | | | | | | | | | | | | | | | | | | | | | | | | | | | | | | | | | | | | | | | | | | | | | | | | | | | | | |  |  |
| Extracorporeal dialysis in neonatal hyperammonemia: modalities and prognostic indicators [26]. | | | Picca S et al. | | 2001 | | | Italy | | |  | | | | | *5.8 (2-21) | | |  | | | 10 | | | | 8 | | | *31(5.5-60) | | | | 1419.5 (729- 4531) | | | | 114 (49- 361) | | | |  | | | |  | | | |  | | | |  | | |  | | | | *3.4±3.6 | | |  | | | | 3 | | | |  | | | | | | | | | | | | | | | | | | | | | | | | | | | | | | | | | | | | | | | | | | | | | | | | | | | | | | | | | | | 2 | | | | | | | *8.25 (7.5-9) | | | | | | | *1412.5 (1099 - 1726) | | | | | | | | *292.5 (224-361) | | | | | | |  | | | | | | | |  | | | | | | |  | | | | | | | | *1.6 ± 0.4 h | | | | | | |  | | | | | | |  | | | | | | | |  | | | | | | | | |  | | | | | | | | | 1 | | | | | | | | |
| Short-term results of continuous venovenous haemodiafiltration versus peritoneal dialysis in 40 neonates with inborn errors of metabolism[27] | | | Celik M et al. | | 2019 | | | Turkey | | | CKRT:3 [2–6] ,PD: 4 [3–8] | | | | |  | | | CKRT: 6 [4–11], PD: 7 [5–8.5] | | | 40 | | | | 11 | | | 48 [24–72] | | | |  | | | |  | | | |  | | | | 44 [27–52] | | | |  | | | |  | | |  | | | | 6 [6–9.5] | | |  | | | | 5 | | | | 29 | | | | | 60 [24–120] | | | | 970.1 [635.35 - 1611.57] | | | |  | | | | |  | |  | | | | | | 14 [6–30] | | |  | | | | | | 3600 (2440 - 4667) | | | |  | | | |  | | | |  | | | 11 | | | | | | |  | | | | | | | | | | | | | | | | | | | | | | | | | | | | | | | | | | | | | | | | | | | | | | | | | | | | | | | | | | | | | | | | | | | | | | | | | | | | | | | | | | | | | | | | | | | | | | | | | | | | |  |  |
| High prevalence of neonatal presentation in Korean patients with citrullinemia type 1, and their shared mutations[28] | | | Lee BH et al. | | 2012 | | | Korea | | | 6 (1-5219.5) | | | | |  | | |  | | | 13 | | | | 6 | | |  | | | |  | | | |  | | | |  | | | |  | | | |  | | | |  | | |  | | | | *4.4±1.5 (2.5-6 h) | | |  | | | |  | | | | 7 | | | | |  | | | |  | | | |  | | | | |  | | *278.4 ± 271.2 (96–672) (<250 μmol/L) | | | | | |  | | | *16.3± 10.9 (5–27) | | | | | |  | | | |  | | | |  | | | |  | | |  | | | | | | |  |  |  |  |  |  |  |  |  |  |  |  |  |  |  |  |  |  |  |  |  |  |  |  |  |  |  |  |  |  |  |  |  |  |  |  |  |  |  |  |  |  |  |  |  |  |  |  |  |  |  |  |  |  |  |  |  |  |  |  |  |  |  |  |  |  |  |  |  |  |  |  |  |  |  |  |  |  |  |  |  |  |  |  |  |  |  |  |  |  |  |  |  |  |  |  |  |  |  |  |  |  |  |
| Continuous venovenous haemodialysis (CVVHD) and continuous peritoneal dialysis (CPD) in the acute management of 21 children with inborn errors of metabolism[29] | | | Arbeiter AK et al. | | 2009 | | | Germany | | |  | | | | | CKRT: *4.1 ± 2.5, PD: *4.5 ± 3.0 | | |  | | | 21 | | | | 17 | | | *42.0±30.4 | | | | *1717±1643 | | | | *274±785 | | | |  | | | | 22.4±18.1 | | | |  | | | |  | | |  | | | | *4.7±2.5 | | |  | | | | 2 | | | | 4 | | | | | *59.4 ± 87.2 | | | | *528.5 ± 303.6 | | | | *509.1 ± 952.4 | | | | |  | | *35.0 ± 24.1 | | | | | |  | | | *13.5 ± 6.2 | | | | | |  | | | |  | | | |  | | | |  | | | 2 | | | | | | |  |  |  |  |  |  |  |  |  |  |  |  |  |  |  |  |  |  |  |  |  |  |  |  |  |  |  |  |  |  |  |  |  |  |  |  |  |  |  |  |  |  |  |  |  |  |  |  |  |  |  |  |  |  |  |  |  |  |  |  |  |  |  |  |  |  |  |  |  |  |  |  |  |  |  |  |  |  |  |  |  |  |  |  |  |  |  |  |  |  |  |  |  |  |  |  |  |  |  |  |  |  |  |
| Peritoneal dialysis in neonates with inborn errors of metabolism: is it really out of date? [30] | Pela I et al. | | | | | 2008 | | | Italy | | |  | | | *3.2 (2.4-4.6) | | | | |  | | | 7 | | | |  | | | | | | | | | | | | | | | | | | | | | | | | | | | | | | | | | | | | | | | | | | | | | | 7 | | | | 120 (5-360) | | | | | | | |  | | | | | | |  | | | | | | |  | | | | | | | | | | 27 (22-96) | | | | | | |  | | | | | | | |  | | | | | | | |  | | | | | | | |  | | | | | | |  | | | | | | | |  | | | | | | | 3 | | | | | | | |  | | | | | | | | | | | | | | | | | | | | | | | | | | | | | | | | | | | | | | | | | | | | | | | | | | | | | | | | | |  |  |  |
| Dialysis in neonates with inborn errors of metabolism [31] | Schaefer F et al. | | | | | 1999 | | | Germany | | |  | | | CKRT: *7.6 (4-12), PD: *21.4 (4-60) | | | | |  | | | 12 | | | | 7 | | | *25±21 | | | |  | | | |  | | | |  | | | |  | | | |  | | | |  | | |  | | | | *7.9 (4.4-15) | | | 2.1 | | | | | | 3 | | | | | 5 | | | | | | *73 ± 35 | | | | | | |  | | | | | | |  | | | | | | | | | |  | | | | | | | |  | | | | | | |  | | | | | | | 6.8 (6.5-7.1) | | | | | | | | |  | | | | | | |  | | | | | | | |  | | | | | | | 24 (16-36) | | | | | | | | 1 | | | | | | |  | | | | | | | | | | | | | | | | | | | | | | | | | | | | | | | | | | | | | | | | | | | | | | | | | | | | | | | | | |
| Continuous veno-venous hemodiafiltration in neonates with maple syrup urine disease [32] | Deger I et al. | | | | | 2022 | | | Turkey | | |  | | | CKRT:13 (7–28) , PD: 9 (7–28) | | | | |  | | | 16 | | | | 11 | | | 36 (12–48) | | | |  | | | |  | | | |  | | | |  | | | | 3000 (1736–5796) | | | | 198 (20-721) | | | 2.56% (1.75-7.6) | | | |  | | |  | | | | | | 0 | | | | | 5 | | | | | | 120 (48–156) | | | | | | |  | | | | | | |  | | | | | | | | | |  | | | | | | | |  | | | | | | |  | | | | | | |  | | | | | | | | | 3600 (2440–4667) | | | | | | | 600 (250-967) | | | | | | | | 0.78 (0.54-1.83) | | | | | | |  | | | | | | | | 0 | | | | | | |  |  |  |  |  |  |  |  |  |  |  |  |  |  |  |  |  |  |  |  |  |  |  |  |  |  |  |  |  |  |  |  |  |  |  |  |  |  |  |  |  |  |  |  |  |  |  |  |  |  |  |  |  |  |  |  |  |  |
| Multisite Retrospective Review of Outcomes in Renal Replacement Therapy for Neonates with Inborn Errors of Metabolism [33] | Ames EG et al. | | | | | 2022 | | | United States | | | 3 [2–4] | | |  | | | | |  | | | 51 | | | | 21 | | |  | | | |  | | | |  | | | |  | | | |  | | | |  | | | |  | | |  | | | |  | | |  | | | | | | 4 | | | | | 1 | | | | | |  | | | | | | |  | | | | | | |  | | | | | | | | | |  | | | | | | | |  | | | | | | |  | | | | | | |  | | | | | | | | |  | | | | | | |  | | | | | | | |  | | | | | | |  | | | | | | | | 0 | | | | | | | 29 | | |  | | | | |  | | | |  | | | | |  | | |  | | | |  | | |  | | | | |  | | | |  | | | | |  | | | |  | | | | | 14 | | | | | | | |
| Continuous Renal Replacement Therapy for Treatment of Severe Attacks of Inborn Errors of Metabolism [34] | Yetimakman AF et al. | | | | | 2019 | | | Turkey | | |  | | | 243.3 (76–1581.7) | | | | |  | | | 25 | | | | 25 | | | 43.3 (6-144) | | | |  | | | |  | | | |  | | | |  | | | |  | | | |  | | |  | | | |  | | |  | | | | | | 10 | | | | |  | | | | | | | | | | | | | | | | | | | | | | | | | | | | | | | | | | | | | | | | | | | | | | | | | | | | | | | | | | | | | | | | | | | | | | | | | | | | | | | | | | | | | | | | | | | | | | | | | |  | | | | | | | | | | | | | | | | | | | | | | | | | | | | | | | | | | | | | | | | | | | | | | | | | | | | | | | | | |
| Treatment of maple syrup urine disease with high flow hemodialysis in a neonate [35] | Aygun F et al. | | | | | 2019 | | | Turkey | | |  | | | *12.3 (5-19) | | | | |  | | | 4 | | | | 4 | | | 10 (6-18) | | | |  | | | |  | | | |  | | | |  | | | |  | | | |  | | | 8.9% (5.5-12.8) | | | |  | | |  | | | | | | 0 | | | | |  |  |  |  |  |  |  |  |  |  |  |  |  |  |  |  |  |  |  |  |  |  |  |  |  |  |  |  |  |  |  |  |  |  |  |  |  |  |  |  |  |  |  |  |  |  |  |  |  |  |  |  |  |  |  |  |  |  |  |  |  |  |  |  |  |  |  |  |  |  |  |  |  |  |  |  |  |  |  |  |  |  |  |  |  |  |  |  |  |  |  |  |  |  |  |  |  |  |  |  |  |  |  |  |  |  |  |  |  |  |  |  |  |  |  |  |  |  |  |  |  |  |  |  |  |  |  |  |  |  |  |  |  |  |  |  |  |  |  |  |  |  |  |  |  |  |  |  |  |  |  |  |  |  |  |  |
| Continuous venovenous hemofiltration in neonates with hyperammonemia. A case series. [36] | María FCS et al. | | | | | 2018 | | | Chile | | |  | | | *10 (3-23) | | | | |  | | | 6 | | | | 6 | | | 49.5 | | | | 976.5 (701.7-1818.4) | | | |  | | | |  | | | |  | | | |  | | | |  | | |  | | | |  | | |  | | | | | | 2 | | | | |  |  |  |  |  |  |  |  |  |  |  |  |  |  |  |  |  |  |  |  |  |  |  |  |  |  |  |  |  |  |  |  |  |  |  |  |  |  |  |  |  |  |  |  |  |  |  |  |  |  |  |  |  |  |  |  |  |  |  |  |  |  |  |  |  |  |  |  |  |  |  |  |  |  |  |  |  |  |  |  |  |  |  |  |  |  |  |  |  |  |  |  |  |  |  |  |  |  |  |  |  |  |  |  |  |  |  |  |  |  |  |  |  |  |  |  |  |  |  |  |  |  |  |  |  |  |  |  |  |  |  |  |  |  |  |  |  |  |  |  |  |  |  |  |  |  |  |  |  |  |  |  |  |  |  |  |
| Renal Replacement Therapy in the Critically Ill Child [37] | Westrope CA et al. | | | | | 2018 | | | United Kingdom | | |  | | |  | | | | |  | | | 145 | | | | 137 | | |  | | | |  | | | |  | | | |  | | | |  | | | |  | | | |  | | |  | | | |  | | |  | | | | | | 42 | | | | | 8 | | | | | |  | | | | | | |  | | | | | | |  | | | | | | | | | |  | | | | | | | |  | | | | | | |  | | | | | | |  | | | | | | | | |  | | | | | | |  | | | | | | | |  | | | | | | |  | | | | | | | | 5 | | | | | | |  |  |  |  |  |  |  |  |  |  |  |  |  |  |  |  |  |  |  |  |  |  |  |  |  |  |  |  |  |  |  |  |  |  |  |  |  |  |  |  |  |  |  |  |  |  |  |  |  |  |  |  |  |  |  |  |  |  |
| The impact of continuous renal replacement therapy for metabolic disorders in infants. [38] | Aygun F et al. | | | | | 2018 | | | Turkey | | |  | | | *167.3 ± 225.1 (2- 547.5) | | | | |  | | | 14 | | | | 14 | | | *16.6 ± 15.6 h | | | |  | | | |  | | | |  | | | |  | | | |  | | | |  | | |  | | | |  | | |  | | | | | | 2 | | | | |  | | | | | | | | | | | | | | | | | | | | | | | | | | | | | | | | | | | | | | | | | | | | | | | | | | | | | | | | | | | | | | | | | | | | | | | | | | | | | | | | | | | | | | | | | | | | | | | | | |  |  |  |  |  |  |  |  |  |  |  |  |  |  |  |  |  |  |  |  |  |  |  |  |  |  |  |  |  |  |  |  |  |  |  |  |  |  |  |  |  |  |  |  |  |  |  |  |  |  |  |  |  |  |  |  |  |  |
| Duration of extracorporeal therapy in acute maple syrup urine disease: a kinetic model [39] | Phan V et al. | | | | | 2006 | | | Canada | | | *2737.5 (474.5–5110) | | |  | | | | |  | | | 7 | | | | 4 | | | 17.1 ± 6.0 | | | |  | | | |  | | | |  | | | |  | | | | *1598 (1073- 2086) | | | | *726.9 (622-1097) | | |  | | | |  | | |  | | | | | | 1 | | | | |  |  |  |  |  |  |  |  |  |  |  |  |  |  |  |  |  |  |  |  |  |  |  |  |  |  |  |  |  |  |  |  |  |  |  |  |  |  |  |  |  |  |  |  |  |  |  |  |  |  |  |  |  |  |  |  |  |  |  |  |  |  |  |  |  |  |  |  |  |  |  |  |  |  |  |  |  |  |  |  |  |  |  |  |  |  |  |  |  |  |  |  |  |  |  |  |  |  | 3 | | | *5.4 ± 0.6 (4.5-6 | | | | | *1667.8 (1345-1964) | | | | *379.75( 286-439) | | | | |  | | |  | | | |  | | |  | | | | |  | | | |  | | | | |  | | | |  | | | | | 0 | | | | | | | |
| Acute hemodialysis for hyperammonemia in small neonates. [40] | | Rajpoot DK et al. | | | | | | | | | | | | 2004 | | | | United States | | | | | | *3.2 (0.3-10) | | | | | | | |  | | | |  | | | | 4 | | |  | | | | | | | | | | | | | | | | | | | | | | | | | | | | | | | | | | | | | | | | | | | | | | | | | | | | | | | | | | | | |  | | | | | | | | | | | | | | | | | | | | | | | | | | | | | | | | | | | | | | | | | | | | | | | | | | | | | | | | | | | | | | | | | | | | | | | | | 4 | | | | | | 5.5 (4-11) | | | *595 (500-700) | | | | | *138 (128-160) | | | | | | |  |  | | | |  | | | |  |  | | | |  | | | |  |  | | | | 0 | | | | | | | | | |
| Combined nutritional support and continuous extracorporeal removal therapy in the severe acute phase of maple syrup urine disease [41] | | Jouvet P et al. | | | | | | | | | | | | 2001 | | | | France | | | | | | 912.5 (11- 4745) | | | | | | | |  | | | |  | | | | 12 | | | 12 | | | |  | | | | |  | | | | | | |  | | | | | | |  | |  | | | | | | *2581 (1073-3818) | | | | | | | | *771 (196- 1275) | | | | |  | | | | | | |  | | | |  | | | | 1 | | | | |  | | | | | | | | | | | | | | | | | | | | | | | | | | | | | | | | | | | | | | | | | | | | | | | | | | | | | | | | | | | | | | | | | | | | | | | |  | | | | | | | | | | | | | | | | | | | | | | | | | | | | | | | | | | | | | | | | | | | | | |  |  |  |  |  |  |
| Application of continuous renal replacement therapy in the treatment of neonates with inherited metabolic diseases [42] | | Hu Y et al. | | | | | | | | | | | | 2021 | | | | China | | | | | |  | | | | | | | | *5.7±2.0 | | | |  | | | | 11 | | | 11 | | | | *44±14 | | | | | *1094±314 | | | | | | | *134±88 | | | | | | |  | |  | | | | | |  | | | | | | | |  | | | | |  | | | | | | |  | | | |  | | | | 3 | | | | |  |  |  |  |  |  |  |  |  |  |  |  |  |  |  |  |  |  |  |  |  |  |  |  |  |  |  |  |  |  |  |  |  |  |  |  |  |  |  |  |  |  |  |  |  |  |  |  |  |  |  |  |  |  |  |  |  |  |  |  |  |  |  |  |  |  |  |  |  |  |  |  |  |  |  |  |  |  |  |  |  |  |  |  |  |  |  |  |  |  |  |  |  |  |  |  |  |  |  |  |  |  |  |  |  |  |  |  |  |  |  |  |  |  |  |  |  |  |  |  |  |  |  |  |
| Efficacy and safety of intermittent hemodialysis in infants and young children with inborn errors of metabolism. [43] | | Tsai IJ. et al. | | | | | | | | | | | | 2014 | | | | Taiwan | | | | | |  | | | | | | | | (7-2555) | | | |  | | | | 7 | | |  | | | | | | | | | | | | | | | | | | | | | | | | | | | | | | | | | | | | | | | | | | | | | | | | | | | | | | | | | | | | |  | | | | | | | | | | | | | | | | | | | | | | | | | | | | | | | | | | | | | | | | | | | | | | | | | | | | | | | | | | | | | | | | | | | | | | | | | 7 | | | | | |  | | |  | | | | |  | | | | | | |  |  | | | |  | | | |  |  | | | |  | | | |  |  | | | | 1 | | | | | | | | | |
| The implementation of neonatal peritoneal dialysis in a clinical setting. [44] | | Unal S. et al. | | | | | | | | | | | | 2012 | | | | Turkey | | | | | |  | | | | | | | |  | | | |  | | | | 10 | | |  |  |  |  |  |  |  |  |  |  |  |  |  |  |  |  |  |  |  |  |  |  |  |  |  |  |  |  |  |  |  |  |  |  |  |  |  |  |  |  |  |  |  |  |  |  |  |  |  |  |  |  |  |  |  |  |  |  |  |  |  | 10 | | | | | | | |  | | | | |  | | | | |  | | | | | | |  | | | |  | | | | | |  | | | | | |  | | |  | | | | |  | | | | | | |  | | | | |  | | | | | | | 4 | | | | |  | | | | | | | | | | | | | | | | | | | | | | | | | | | | | | | | | | | | | | | | | | | | | | | | | | | | | |
| Continuous renal replacement therapy for non-renal indications: experience in children [45] | | Kornecki A. et al. | | | | | | | | | | | | 2002 | | | | Israel | | | | | |  | | | | | | | | *12 (8-16) | | | |  | | | | 3 | | | 3 | | | | *13 (4.5-24) | | | | |  | | | | | | |  | | | | | | |  | |  | | | | | |  | | | | | | | |  | | | | |  | | | | | | |  | | | |  | | | |  | | | | |  | | | | | | | | | | | | | | | | | | | | | | | | | | | | | | | | | | | | | | | | | | | | | | | | | | | | | | | | | | | | | | | | | | | | | | | |  | | | | | | | | | | | | | | | | | | | | | | | | | | | | | | | | | | | | | | | | | | | | | |  |  |  |  |  |  |
| Continuous renal replacement therapy and plasma exchange in newborns and infants[46] | | Ponikvar R. et al. | | | | | | | | | | | | 2002 | | | | Slovenija | | | | | |  | | | | | | | |  | | | |  | | | | 2 | | | 2 | | | | *9 (7.5-9.5) | | | | | *699 (128-1401) | | | | | | | *166 (47-347) | | | | | | |  | |  | | | | | |  | | | | | | | |  | | | | |  | | | | | | |  | | | |  | | | |  | | | | |  |  |  |  |  |  |  |  |  |  |  |  |  |  |  |  |  |  |  |  |  |  |  |  |  |  |  |  |  |  |  |  |  |  |  |  |  |  |  |  |  |  |  |  |  |  |  |  |  |  |  |  |  |  |  |  |  |  |  |  |  |  |  |  |  |  |  |  |  |  |  |  |  |  |  |  |  |  |  |  |  |  |  |  |  |  |  |  |  |  |  |  |  |  |  |  |  |  |  |  |  |  |  |  |  |  |  |  |  |  |  |  |  |  |  |  |  |  |  |  |  |  |  |  |

**Data expressed as median (IQR) or mean** ± **SD**

***median (min-max)**

**Table S4: Indications for KRT and Dialysis Prescription**

| **Studies** | **Indications for initiating KRT in IEM patients** | **Qb: Qd (range)** |
| --- | --- | --- |
| Eminoğlu FT et al., 2022^9^ | Persistently high blood ammonia levels (≥ 500 μmol/L); blood ammonia levels of > 250 μmol/L in the presence of moderate encephalopathy high blood leucine levels (≥ 1500 μmol/L); blood leucine levels of < 1500 μmol/L in the presence of rapidly deteriorating neurological status, moderate to severe encephalopathy and seizures, or, despite medical treatment, ongoing persistent metabolic acidosis | Qb: 4–12 mL/kg/min  Qd: 4 L/1.73 m^2^/h (at onset)  Qd: 2 L/1.73 m^2^/h (at normalization) |
| Akduman H et al., 2020^8^ | ammonium level > 500 µmol/L, metabolic acidosis that is unresponsive to medications, or had signs of encephalopathy | Qb: 8–12 mL/kg/min  Qd: 2000 mL/1.73 m^2^ per hour |
| Abily-Donval L et al., 2020^14^ | Ammonia levels exceeded 500 mmol/L, or if the ammonia levels exceeded 250 mmol/L with signs of metabolic encephalopathy, or if the ammonia levels increased quickly despite introduction of metabolite scavengers or in case of very early onset of disease | Qb: 9.4 mL/ kg/min (6.7–19.9)  Qd: 204.7 mL/kg/hour (24.4–917.4)  (Range) |
| Aygun F et al., 2019^32^ | Indications for CKRT included fluid overload, electrolyte imbalance, metabolic acidosis, inherited metabolic diseases metabolic disease, and intoxication/ | Qb: 5–20 mL/kg/min  Qd: 2- 12.9 L/1.73 m^2^/h. |
| Kim JY et al., 2019^15^ | Ammonia level >200 µmol/L and/or neurologic symptoms regardless of medical management | Qb (initial) : 8.6 (7.2–14.8) mL/kg/min  Qb (peak): 9.7 (8.2–15.5) mL/kg/min  Qd (initial): 150 (100–400) mL/hour  Qd (peak): 225 (100–600) mL/hour |
| Demirkol D et al., 2019^10^ | - | Qb: 6.3 (4.3– 8.2) mL/kg/min  Qd: 2,162 (2,000–4,016) mL/h/1.73 m^2^ |
| Mok TYD et al., 2018^13^ | - | Qb: 3-10 mL/kg/min |
| Demirkol D et al., 2016^11^ | Late diagnosis, or when neurological signs worsened | Qb: 4.3 (3–7.5) ml/kg/min  Qd: 2,163 (1,750– 3,200) ml/1.73 m^2^/h |
| Westrope C et al., 2010^12^ | Ammonia level (>200 µmol/l) | Qb: (5.7- 24.0) mL/kg/min |
| Hiroma T et al., 2002^33^ | Respiratory failure, lethargy, hypotonia. The ammonia level on admission was 260–1790 g/dL. | Qb: 20 to 30 mL/min  Qd: 1 to 2 L/hour |
| Celik M et al., 2019^19^ | Blood ammonia level >1000 μg/dl, or 4 hours with a blood ammonia level of 500–1000 μg/dl, | Qd (day 1): 10-20 ml/kg  Qd (day 2): 30 ml/kg |
| Picca S et al., 2015^34^ | Dialysis was started at the decision of local physicians when ammonium levels were considered too high to be treated only with medical measures or if patients failed to respond rapidly to medical treatment. | - |
| Bilgin L et al., 2014^18^ | PD was indicated with increasing plasma ammonia levels or values persistently above 500 μmol/L. | - |
| Eisenstein I et al., 2022^17^ | HD was initiated if, by 4 h of conservative treatment, repeated plasma ammonia levels exceeded 500 μmol/L, or the presence of neurologic deterioration | - |
| Lai YC et al., 2007^25^ | Failure of medical management and acute neurologic deterioration | Qb: 16 (16-100) ml/min  Qd: 999 mL/hour |
| Picca S et al., 2004^24^ | Failure to respond to pharmacological therapy alone | Qb (CAVHD): 14 (8-31) ml/min  Qb (CVVHD): 20-40 ml/min  Qb (HD): 10-15 ml/min  Qd (CAVHD): 8.3 ml/min  Qd (CVVHD): 8.3-33.3 ml/min  Qd (HD): 500 ml/min |
| Celik M et al., 2019^35^ | Ammonia level on admission was > 1000 μg/ dL, or 500–1000 μg/dL and then kept increasing in the fourth hour of medical treatment. | Qb: 10 ml/kg/min  Qd: 2000 mL/1.73 m^2^/h |
| Lee BH et al., 2012^20^ | Hyperammonemic encephalopathy | - |
| Arbeiter AK et al., 2009^21^ | Failure of conservative treatment | Qb: 9.8 (3.7–22.7) ml/kg/min  Qd: 3925 mL/1.73 m^2^/h |
| Pela I et al., 2008^36^ | Plasma ammonia levels above 1,000 μg/dl or when, despite pharmacological and dietetic treatment, plasma ammonia levels remained over 700 μg/dl. |  |
| Schaefer F et al., 1999^22^ | Elevated ammonia level and failure to respond to nutritional and medical management | Qb: 10-30 ml/min  Qd: 1 to 5 L/hour |
| Deger I et al., 2022^23^ | Patient developing coma, gastrointestinal intolerance, worsening clinical condition, inability to feed | Qb: 10-20 mL/kg/min  Qd: 2000 mL/1.73 m^2^/h |
| Yetimakman AF et al., 2019^39^ | CKRT was initiated based on ongoing acidosis and/or clinical needs for each patient including in the first 24 hours for acute encephalopathy, and as soon as possible for high ammonia levels. | Qb: 4-12 ml/kg/min  Qd: 3-5 L/1.73 m^2^/h |
| Aygun F et al., 2019^40^ |  | Qd: 4120 - 9830 ml/h/1.73m^2^. |
| María FCS et al., 2018^41^ | Decision of the PICU team guided by response to medical management in the first 24 hours, progressive neurological involvement, or increased blood ammonia levels (>400 μmol/L) | Qb: 8-20 ml/kg/min  Qd: 2000-2500 ml/1.73 m^2^ /hour. |
| Aygun F et al., 2018^43^ | - | Qb: 10 ml/kg/min  Qd: 4042 -12,900 ml/1.73 m^2^ /hour. |
| Rajpoot DK et al., 2004^48^ | All patients approaching a plasma ammonia level of 500 mmol/l with clinical symptoms were considered for emergency HD. | Qb: 15–30 ml/min |

**Table S5: Newcastle Ottawa Risk of Bias Quality Assessment**

| Cohort Studies |  | Selection | | | | Comparability | Exposure | | |  |
| --- | --- | --- | --- | --- | --- | --- | --- | --- | --- | --- |
| First Author | Year | Representativeness of exposed cohort | Selection of the non-exposed cohort | Ascertainment of exposure | Demonstration that outcome of interest was not present at start of study | Comparability of cohorts based on the design or analysis | Assessment of outcome | Was follow-up long enough for outcomes to occur | Adequacy of follow up cohorts | Quality Score |
| Eminoğlu FT ^9^ | 2022 | 1 | 1 | 1 | 1 | 1 | 1 | 1 | 1 | 8/9 |
| Akduman H ^8^ | 2020 | 1 | 1 | 1 | 1 | 1 | 1 | 1 | 1 | 8/9 |
| Abily-Donval L ^14^ | 2020 | 1 | 1 | 1 | 1 | 2 | 1 | 1 | 1 | 9/9 |
| Aygun F ^32^ | 2019 | 1 | 1 | 1 | 1 | 2 | 1 | 1 | 1 | 9/9 |
| Kim JY ^15^ | 2019 | 1 | 1 | 1 | 1 | 1 | 1 | 1 | 1 | 8/9 |
| Demirkol D ^10^ | 2019 | 1 | 1 | 1 | 1 | 1 | 1 | 1 | 1 | 8/9 |
| Mok TYD ^13^ | 2018 | 1 | 1 | 1 | 1 | 2 | 1 | 1 | 1 | 9/9 |
| Demirkol D^11^ | 2016 | 1 | 1 | 1 | 1 | 1 | 1 | 1 | 1 | 8/9 |
| Westrope C^12^ | 2010 | 1 | 1 | 1 | 1 | 2 | 1 | 1 | 1 | 9/9 |
| Hiroma T ^33^ | 2002 | 1 | 1 | 1 | 1 | 2 | 1 | 1 | 1 | 9/9 |
| Celik M ^19^ | 2019 | 1 | 1 | 1 | 1 | 2 | 1 | 1 | 1 | 9/9 |
| Picca S ^34^ | 2015 | 1 | 1 | 1 | 1 | 2 | 1 | 1 | 1 | 9/9 |
| Bilgin L ^18^ | 2014 | 1 | 1 | 1 | 1 | 1 | 1 | 1 | 1 | 8/9 |
| Robinson JR ^16^ | 2018 | 1 | 1 | 1 | 1 | 2 | 1 | 1 | 1 | 9/9 |
| Eisenstein I ^17^ | 2022 | 1 | 1 | 1 | 1 | 1 | 1 | 1 | 1 | 8/9 |
| Lai YC ^25^ | 2007 | 1 | 1 | 1 | 1 | 1 | 1 | 1 | 1 | 8/9 |
| Picca S ^24^ | 2004 | 1 | 1 | 1 | 1 | 1 | 1 | 1 | 1 | 8/9 |
| Celik M ^35^ | 2019 | 1 | 1 | 1 | 1 | 1 | 1 | 1 | 1 | 8/9 |
| Lee BH ^20^ | 2012 | 1 | 1 | 1 | 1 | 1 | 1 | 1 | 1 | 8/9 |
| Arbeiter AK^21^ | 2009 | 1 | 1 | 1 | 1 | 1 | 1 | 1 | 1 | 8/9 |
| Pela I^36^ | 2008 | 1 | 1 | 1 | 1 | 2 | 1 | 1 | 1 | 9/9 |
| Schaefer F^22^ | 1999 | 1 | 1 | 1 | 1 | 1 | 1 | 1 | 1 | 8/9 |
| Deger I ^23^ | 2022 | 1 | 1 | 1 | 1 | 1 | 1 | 1 | 1 | 8/9 |
| Ames EG^37^ | 2022 | 1 | 1 | 1 | 1 | 1 | 1 | 1 | 1 | 8/9 |
| Yetimakman AF ^39^ | 2019 | 1 | 1 | 1 | 1 | 1 | 1 | 1 | 1 | 8/9 |
| Aygun F ^40^ | 2019 | 1 | 1 | 1 | 1 | 1 | 1 | 1 | 1 | 8/9 |
| María FCS ^41^ | 2018 | 1 | 1 | 1 | 1 | 2 | 1 | 1 | 1 | 9/9 |
| Westrope CA ^42^ | 2018 | 1 | 1 | 1 | 1 | 1 | 1 | 1 | 1 | 8/9 |
| Aygun F ^43^ | 2018 | 1 | 1 | 1 | 1 | 1 | 1 | 1 | 1 | 8/9 |
| Phan V ^46^ | 2006 | 1 | 1 | 1 | 1 | 1 | 1 | 1 | 1 | 8/9 |
| Rajpoot DK^48^ | 2004 | 1 | 1 | 1 | 1 | 2 | 1 | 1 | 1 | 9/9 |
| Jouvet P ^49^ | 2001 | 1 | 1 | 1 | 1 | 1 | 1 | 1 | 1 | 8/9 |
| Hu Y et al. | 2021 | 1 | 1 | 1 | 1 | 1 | 1 | 1 | 1 | 8/9 |
| Cavagnaro Santa María F. et al. | 2018 | 1 | 1 | 1 | 1 | 2 | 1 | 1 | 1 | 9/9 |
| Tsai IJ. et al. | 2014 | 1 | 1 | 1 | 1 | 1 | 1 | 1 | 1 | 8/9 |
| Unal S. et al. | 2012 | 1 | 1 | 1 | 1 | 2 | 1 | 1 | 1 | 9/9 |
| Kornecki A. et al. | 2002 | 1 | 1 | 1 | 1 | 1 | 1 | 1 | 1 | 8/9 |
| Ponikvar R. et al. | 2002 | 1 | 1 | 1 | 1 | 1 | 1 | 1 | 1 | 8/9 |

**Table S6. IEM disorders among neonates/ children receiving KRT included across gathered studies**

| Author & Year | IEM Sample Size (n) | Urea Cycle Disorders (UCD) | Organic Acidemias (OA) | Other IEM | Unknown diagnosis |
| --- | --- | --- | --- | --- | --- |
| Eminoğlu FT et al., 2022^9^ | 22 | 8 (36.4%) | 14 (63.6%):  9 MSUD  5 not specified |  | - |
| Akduman H et al., 2020^8^ | 8 | 5 (62.5%) | 1 MMA (12.5%)  2 MSUD (25%) |  | - |
| Abily-Donval L et al., 2020^14^ | 14 | 9 (64.2%): | 2 (14.2%) |  | 3 (21.4%) |
| Aygun F et al., 2019^32^ | 36 | 12 (33.3%) | 24 (66.7%):  17 MSUD  7 not specified |  | - |
| Kim JY et al., 2019^15^ | 12 | 10 (83.3%):  4 OTCD  2 CPSD  4 ASS | 2 PA (16.7%) | - | - |
| Demirkol D et al., 2019^10^ | 10 | 9 (90%):  4 CPSD  2 ASS  2 ASL  1 NAGS | 1 PA (10%) | - | - |
| Mok TYD et al., 2018^13^ | 3 | 1 OTCD (33.3%) | 2 (66.7%):  1 MMA  1 not specified | - | - |
| Demirkol D et al., 2016^11^ | 14 | - | 14 MSUD (100%) |  | - |
| Westrope C et al., 2010^12^ | 14 | 8 (57.1%):  2 OTCD  1 CPSD  5 ASS | 6 (42.9%):  2 MMA  4 PA | - | - |
| Hiroma T et al., 2002^33^ | 4 | 1 CPSD (25%) | 1 MMA (25%) | 2 PCD (50%) | - |
| Celik M et al., 2019^19^ | 14 | 8 (57.1%):  1 OTCD  6 ASS  1 ASL | 6 (42.9%):  2 MMA  3 PA  1 not specified | - | - |
| Picca S et al., 2015^34^ | 23 | 12 (52.2%):  1 CPSD  4 ASS  7 ASL | 11 (47.8%):  5 MMA  6 PA | - | - |
| Bilgin L et al., 2014^18^ | 9 | 2 ASS (22.2%) | 7 (77.8%):  3 MSUD  2 MMA  2 PA | - | - |
| Robinson JR et al., 2018^16^ | 13 | 10 (76.9%) | 1 (7.7%) | - | 2 (15.4%) |
| Eisenstein I et al., 2022^17^ | 20 | 8 (40%):  2 OTCD  2 CPSD  1 ASL  3 ASS | 10 (50%):  7 MSUD  1 MMA  1 PA  1 IVA | 1 E3 (5%) | 1 (5%) |
| Lai YC et al., 2007^25^ | 6 | 2 (33.3%)  1 OTCD  1 CPSD | 4 (66.6.7%)  1 MSUD  3 MMA | - | - |
| Picca S et al., 2004^24^ | 10 | 5 (50%):  2 CPSD  2 ASS  1 ASL | 5 (50%):  2 MMA  3 PA | - | - |
| Celik M et al., 2019^35^ | 40 | 21 (50%):  5 OTCD  2 CPSD  10 ASS  3 ASL  1 unspecified | 19 (42.5%):  5 MSUD  7 MMA  5 PA  2 unspecified | - |  |
| Lee BH et al., 2012^20^ | 13 | 13 ASS (100%) | - | - | - |
| Arbeiter AK et al., 2009^21^ | 21 | 15 (71.4%):  3 OTCD  3 CPSD  8 ASS  1 ARG | 4 (19%):  2 MMA  1 PA  1 GA-II | - | 2 (9.5%) |
| Pela I et al., 2008^36^ | 7 | 3 (42.9%):  1 OTCD  1 CPSD  1 ASL | 4 (57.1%):  1 MMA  3 PA | - | - |
| Schaefer F et al., 1999^22^ | 12 | 5 (41.7%):  2 OTCD  2 CPSD  1 ASL | 7 (58.3%):  4 MSUD  3 PA | - | - |
| Deger I et al., 2022^23^ | 16 | - | 16 MSUD (100%) | - | - |
| Ames EG et al., 2022^37^ | 51 | 43 (84.3%):  26 OTCD  7 CPSD  6 ASS  4 ASL | 8 (15.7%):  3 MMA  5 PA | - | - |
| Yetimakman AF et al., 2019^39^ | 25 | 1 ASS (4%) | 17 (68%):  4 MSUD  9 MMA  4 PA | 6 (24%):  1 MCADD  1 SCADD  4 MC | 1 (4%) |
| Aygun F et al., 2019^40^ | 4 | - | 4 MSUD (100%) | - | - |
| María FCS et al., 2018^41^ | 6 | 3 (50%):  2 OTCD  1 ASL | 3 (50%):  1 MMA  2 PA | - | - |
| Westrope CA et al., 2018^42^ | 145 | - | - | - | 145 not specified (100%) |
| Aygun F et al., 2018^43^ | 14 | 10 (71.4%):  5 OTCD  3 CPSD  2 CPS-2 | 4 MSUD (28.6%) | - | - |
| Phan V et al., 2006^46^ | 7 | - | 7 MSUD (100%) | - | - |
| Rajpoot DK et al., 2004^48^ | 4 | 1 OTCD (25%) | 1 MMA (25%) | - | 2 (50%) |
| Jouvet P et al., 2001^49^ | 12 | - | 12 MSUD (100%) | - | - |
| Hu Y et al., 2021 | 11 | 6 (54.5%)  4 OTCD  2 ASS | 5 (45.5%)  1 MSUD  2 MMA  2 PA | - | - |
| Cavagnaro Santa María F. et al., 2018 | 6 | 3 (50%)  2 OTCD  1 ASL | 3 (50%)  2 PA  1 MMA | - | - |
| Tsai IJ. et al., 2014 | 7 | - | - | - | 7 (100%) |
| Unal S. et al., 2012 | 10 | 2 (20%)  2 ASS | 8 (80%)  3 MSUD  2 MMA  2 PA  1 GA-II | - | - |
| Kornecki A. et al., 2022 | 3 | - | 3 (100%)  3 MSUD | - | - |
| Ponikvar R. et al., 2002 | 2 | - | - | - | 2 (100%) |

**Reported as n (%).**

**Abbreviations**: CPSD, carbamoyl phosphate synthetase 1 deficiency; OTCD, ornithine transcarbamylase deficiency; ASS, argininosuccinate synthetase deficiency (aka citrullinemia); ASL, argininosuccinate lyase deficiency (aka argininosuccinate acidemia); MMA, methylmalonic acidemia; PA, propionic acidemia; GA-II, glutaric aciduria type II; IVA, isovaleric acidemia; MSUD, maple-syrup urine disease; : CPS-2, carbamoyl phosphate synthetase 2 deficiency; SCADD, short-chain acyl-CoA dehydrogenase deficiency; MCADD, medium-chain acyl-CoA dehydrogenase deficiency; MC, mitochondrial cytopathy; NAGS, N-acetyl glutamate synthetase deficiency; E3, E3 dihydrolipoyl dehydrogenase deficiency; PCD, pyruvate carboxylase deficiency, ARG, Argininemia; and IEM, inborn errors of metabolism.

**Table S7. Proportion of IEM disorders in included patients**

| IEM Class | IEM Diagnosis (n) | Proportion of IEM class (%) | Proportion of total IEM (%) |
| --- | --- | --- | --- |
| Urea Cycle Disorders (UCD): 225 | OTCD: 56 | 24.9% | 9.2% |
|  | CPSD: 32 | 14.2% | 5.3% |
|  | ASS: 66 | 29.3% | 10.8% |
|  | ASL: 22 | 9.8% | 3.6% |
|  | CPS-2: 2 | 0.9% | 0.3% |
|  | ARG: 1 | 0.4% | 0.2% |
|  | NAGS: 1 | 0.4% | 0.2% |
|  | UCD not specified: 45 | 20.0% | 7.4% |
| Organic Acidemias (OA): 219 | MSUD: 109 | 43.4% | 17.9% |
|  | MMA: 44 | 17.5% | 7.2% |
|  | PA: 45 | 17.9% | 7.4% |
|  | IVA: 1 | 0.4% | 0.2% |
|  | GA-II: 1 | 0.4% | 0.2% |
|  | OA not specified: 19 | 7.6% | 3.1% |
| Other IEM: 165 | E3: 1 | 0.6% | 0.2% |
|  | PCD: 2 | 1.2% | 0.3% |
|  | MC: 4 | 2.4% | 0.7% |
|  | MCADD: 1 | 0.6% | 0.2% |
|  | SCADD: 1 | 0.6% | 0.2% |
|  | Unknown IEM: 156 | 94.5% | 25.6% |
| Total IEM Patients: 609 |  | |  |

**Abbreviations**: CPSD, carbamoyl phosphate synthetase 1 deficiency; OTCD, ornithine transcarbamylase deficiency; ASS, argininosuccinate synthetase deficiency (aka citrullinemia); ASL, argininosuccinate lyase deficiency (aka argininosuccinate acidemia); MMA, methylmalonic acidemia; PA, propionic acidemia; GA-II, glutaric aciduria type II; IVA, isovaleric acidemia; MSUD, maple-syrup urine disease; CPS-2, carbamoyl phosphate synthetase 2 deficiency; SCADD, short-chain acyl-CoA dehydrogenase deficiency; MCADD, medium-chain acyl-CoA dehydrogenase deficiency; MC, mitochondrial cytopathy; NAGS, N-acetyl glutamate synthetase deficiency; E3, E3 dihydrolipoyl dehydrogenase deficiency; PCD, pyruvate carboxylase deficiency, ARG, Argininemia; and IEM, inborn errors of metabolism.

**Figure S1: PRISMA Flow Chart**

**Identification of studies via databases and registers**

Records removed *before screening*:

Duplicate records removed (n =211 )

Records marked as ineligible by automation tools (n =0 )

Records removed for other reasons (n =0 )

Records identified from*:

Databases (n =1126 )

**Identification**

Records screened

(n =915 )

Records excluded**

(n =815 )

Reports sought for retrieval

(n =100 )

Reports not retrieved

(n =12 )

**Screening**

Reports excluded:

Abstract only (n =14)

Incorrect Study Design (n =13)

Incorrect outcomes measured (n = 22)

Not in English (n=1)

Reports assessed for eligibility

(n =88)

Reports of included studies

(n = 38)

**Included**

**Table S8: PRISMA 2020 Manuscript and Abstract Checklist**

| **Section and Topic** | **Item #** | **Checklist item** | **Location where item is reported** |
| --- | --- | --- | --- |
| **TITLE** | | |  |
| Title | 1 | Identify the report as a literature review. | Page 1 |
| **ABSTRACT** | | |  |
| Abstract | 2 | Provide a structured summary including, as applicable: background; objectives; data sources; study eligibility criteria, participants, and interventions; study appraisal and synthesis methods; results; limitations; conclusions and implications of key findings.  See the [PRISMA 2020 for Abstracts checklist](http://www.prisma-statement.org/Extensions/Abstracts.aspx) for the complete list. | Page 2-3 |
| **INTRODUCTION** | | |  |
| Rationale | 3 | Describe the rationale for the review in the context of existing knowledge, i.e., what is already known about your topic. | Page 4 |
| Objectives | 4 | Provide an explicit statement of the objective(s) or question(s) the review addresses with reference to participants, interventions, comparisons, outcomes, and study design (PICOS). | Page 5-6 |
| **METHODS** | | |  |
| Eligibility criteria | 5 | Specify the inclusion and exclusion criteria for the review and how studies were grouped for the syntheses with study characteristics (e.g., PICOS, length of follow-up) and report characteristics (e.g., years considered, language, publication status) used as criteria for eligibility, giving rationale. | Page 6,7,17 |
| Information sources | 6 | Specify all databases, registers, websites, organisations, reference lists and other sources searched or consulted to identify studies. Specify the date when each source was last searched or consulted. | Page 6 |
| Search strategy | 7 | Present the full search strategies for all databases, registers and websites, including any filters and limits used. | Page 6,18-20 |
| Selection process | 8 | State the process for selecting studies (i.e., screening, eligibility).  Specify the methods used to decide whether a study met the inclusion criteria of the review, including how many reviewers screened each record and each report retrieved, whether they worked independently, and if applicable, details of automation tools used in the process. | Page 6,7,16 |
| Study risk of bias assessment | 11 | Specify the methods used to assess risk of bias in the included studies, including details of the tool(s) used, how many reviewers assessed each study and whether they worked independently, and if applicable, details of automation tools used in the process. | Page 7,8 |
| **RESULTS** | | |  |
| Study selection | 16a | Describe the results of the search and selection process, from the number of records identified in the search to the number of studies included in the review, ideally using a flow diagram. | Page 6,7,16 |
|  | 16b | Cite studies that might appear to meet the inclusion criteria, but which were excluded, and explain why they were excluded. | N/A |
| Study characteristics | 17 | Cite each included study and present its characteristics (e.g., study size, PICOS, follow-up period). | Page 22-25 |
| Risk of bias in studies | 18 | Present assessments of risk of bias for each included study. | Page 26-27 |
| Results of individual studies | 19 | For all outcomes, present, for each study: (a) summary statistics for each group (where appropriate) and (b) an effect estimate and its precision (e.g. confidence/credible interval), ideally using structured tables or plots. | Pages 8-11 |
| **DISCUSSION** | | |  |
| Discussion | 23a | Provide a general interpretation of the results in the context of other evidence. | Page 11-14 |
|  | 23b | Discuss any limitations of the evidence included in the review. | Page 14-15 |
|  | 23c | Discuss any limitations of the review processes used. | Page 14-15 |
|  | 23d | Discuss implications of the results for practice, policy, and future research. | Page 13-14 |
| **OTHER INFORMATION** | | |  |
| Registration and protocol | 24a | Provide registration information for the review, including register name and registration number, or state that the review was not registered. | Page 1 |
|  | 24b | Indicate where the review protocol can be accessed, or state that a protocol was not prepared. | Page 1 |
|  | 24c | Describe and explain any amendments to information provided at registration or in the protocol. | N/A |
| Support | 25 | Describe sources of financial or non-financial support for the review, and the role of the funders or sponsors in the review. | Page 15 |
| Competing interests | 26 | Declare any competing interests of review authors. | Page 15 |
| Availability of data, code, and other materials | 27 | Report which of the following are publicly available and where they can be found: template data collection forms; data extracted from included studies; data used for all analyses; analytic code; any other materials used in the review. | Page 14-15 |

| **Section and Topic** | **Item #** | | **Checklist item** | | **Reported (Yes/No)** | |  |  |
| --- | --- | --- | --- | --- | --- | --- | --- | --- |
| **TITLE** | | | | | | |  | |
| Title | | | 1 | | Identify the report as a systematic review. | | Yes | |
| **BACKGROUND** | | | | | | |  | |
| Objectives | | | 2 | | Provide an explicit statement of the main objective(s)  or question(s) the review addresses. | | Yes | |
| **METHODS** | | | | | | |  | |
| Eligibility criteria | | | 3 | | Specify the inclusion and exclusion criteria for the review. | | Yes | |
| Information sources | | | 4 | | Specify the information sources (e.g. databases, registers)  used to identify studies and the date when each was last searched. | | Yes | |
| Risk of bias | | | 5 | | Specify the methods used to assess risk of bias in the included studies. | | Yes | |
| Synthesis of results | | | 6 | | Specify the methods used to present and synthesise results. | | Yes | |
| **RESULTS** | | | | | | |  | |
| Included studies | | | 7 | | Give the total number of included studies and participants  and summarise relevant characteristics of studies. | | Yes | |
| Synthesis of results | | | 8 | | Present results for main outcomes, preferably indicating the number of included studies and participants for each.  If meta-analysis was done, report the summary estimate and confidence/credible interval.  If comparing groups, indicate the direction of the effect (i.e. which group is favoured). | |  | |
| **DISCUSSION** | | | | | | |  | |
| Limitations of evidence | | | 9 | | Provide a brief summary of the limitations of the evidence included in the review  (e.g. study risk of bias, inconsistency and imprecision). | | Yes | |
| Interpretation | | | 10 | | Provide a general interpretation of the results and important implications. | | Yes | |
| **OTHER** | | | | | | |  | |
| Funding | | | 11 | | Specify the primary source of funding for the review. | | Yes | |
| Registration | | | 12 | | Provide the register name and registration number. | | Yes | |
| **Section and Topic** | **Item #** | | **Checklist item** | | **Reported (Yes/No)** | |  |  |
| **TITLE** |  | |  |  |  |  |  |  |
| Title | 1 | | Identify the report as a systematic review. | | Yes | |  |  |
| **BACKGROUND** |  | |  |  |  |  |  |  |
| Objectives | 2 | | Provide an explicit statement of the main objective(s) or question(s) the review addresses. | | Yes | |  |  |
| **METHODS** |  | |  |  |  |  |  |  |
| Eligibility criteria | 3 | | Specify the inclusion and exclusion criteria for the review. | | Yes | |  |  |
| Information sources | 4 | | Specify the information sources (e.g. databases, registers) used to identify studies  and the date when each was last searched. | | Yes | |  |  |
| Risk of bias | 5 | | Specify the methods used to assess risk of bias in the included studies. | | Yes | |  |  |
| Synthesis of results | 6 | | Specify the methods used to present and synthesise results. | | Yes | |  |  |
| **RESULTS** |  | |  |  |  |  |  |  |
| Included studies | 7 | | Give the total number of included studies and participants and  summarise relevant characteristics of studies. | | Yes | |  |  |
| Synthesis of results | 8 | | Present results for main outcomes, preferably indicating the number of included studies and participants for each.  If meta-analysis was done, report the summary estimate and confidence/credible interval.  If comparing groups, indicate the direction of the effect (i.e. which group is favoured). | |  | |  |  |
| **DISCUSSION** |  | |  |  |  |  |  |  |
| Limitations of evidence | 9 | | Provide a brief summary of the limitations of the evidence included in the review  (e.g. study risk of bias, inconsistency and imprecision). | | Yes | |  |  |
| Interpretation | 10 | | Provide a general interpretation of the results and important implications. | | Yes | |  |  |
| **OTHER** |  | |  |  |  |  |  |  |
| Funding | 11 | | Specify the primary source of funding for the review. | | Yes | |  |  |
| Registration | 12 | | Provide the register name and registration number. | | Yes | |  |  |

**AMSTAR Systematic Review Criteria Checklist:**

| Were the PICO components included? | yes |
| --- | --- |
| Was a prior design provided | yes |
| Did the author explain their selection of study designs for inclusion in the review | yes |
| Did the review authors use a comprehensive literature search strategy? | yes |
| Did the review authors perform study selection in duplicate? | yes |
| Did the review authors perform data extraction in duplicate? | yes |
| Did the review authors provide a list of excluded studies and justify the exclusions? | yes |
| Did the review authors describe the included studies in adequate detail? | yes |
| Satisfactory technique to assess RoB was used? | yes |
| Did the review authors report on the sources of funding for the studies included in the review? | no |
| Appropriate methods for statistical combination of results was used for included studies? | yes |
| Review authors assessed the potential impact of RoB in individual studies on the results of the meta-analysis | yes |
| Did the review authors account for RoB in individual studies when interpreting/discussing the results of the review | yes |
| Did the review authors provide a satisfactory explanation for any heterogeneity observed in the results of the review? | yes |
| Was publication bias investigated and its impact on the results of the review? | yes |
| Total score: | 13/14 |
